# Brain insulin resistance as a driver of proteinopathy in neurodegeneration: from cell-type-specific mechanisms to targeted therapeutics

**DOI:** 10.1186/s40035-026-00573-1

**Published:** 2026-08-03

**Authors:** Man Xiang, Si-Yu Cao, Xue-Heng Sun, Jing-Wen Hu, Ming-Zhe Lv, Jia-Yi Li, Wen Li

**Affiliations:** 1 Laboratory of Research in Parkinson’s Disease and Related Disorders, Institute of Health Science, China Medical University, Shenyang, China; 2 Department of Neurosurgery, Shengjing Hospital of China Medical University, Shenyang, China; 3 Department of Laboratory Medicine, Shengjing Hospital of China Medical University, Shenyang, China; 4 Neural Plasticity and Repair Unit, Department of Experimental Medical Sciences, Lund University, 221 84 Lund, Sweden

**Keywords:** Brain insulin resistance, Alzheimer’s disease, Parkinson’s disease, Amyloid-β, Tau, α-Synuclein

## Abstract

Neurodegenerative diseases are increasingly linked to systemic metabolic dysfunction, with brain insulin resistance (BIR) positioned as a central mediator. Yet translating this insight into effective therapies has proven remarkably difficult. This review argues that BIR-driven neurodegeneration should be interpreted at two distinct but interconnected levels: cell-type-specific disruption of brain homeostasis by BIR, and the direct, mechanistic role of BIR in driving the proteinopathies that define Alzheimer’s and Parkinson’s diseases. We first show how BIR produces distinct functional deficits across neurons, astrocytes, microglia, and oligodendrocytes, impairing synaptic plasticity, metabolic coupling, immunometabolic homeostasis, and myelination, resulting in a cellular milieu that favors proteinopathy. We then map molecular pathways through which BIR directly distrubs the metabolism of amyloid-β, tau, and α-synuclein. We further examine how islet amyloid polypeptide cross-seeds cerebral amyloid pathology, suggesting a direct molecular interaction between the peripheral drivers of BIR and protein aggregation. In this framework, BIR functions not as a passive risk factor, but as an active, upstream driver of proteostatic collapse. Cellular dysfunction combined with proteostatic failure, defines the therapeutic target space. We evaluate interventions accordingly, distinguishing those that primarily restore cellular function from those that enhance protein clearance, and those that achieve both. For each strategy, we assess the translational evidence, critically appraising the barriers that have limited their clinical success, including patient heterogeneity, narrow therapeutic windows, and inadequate central nervous system delivery. By integrating cell-type-specific biology with proteostatic mechanisms and a clinically oriented therapeutic framework, this review aims to provide a foundation for multi-target strategies that address the BIR–neurodegeneration axis at its mechanistic roots.

## Introduction

Neurodegenerative diseases (NDDs) are characterized by the progressive loss of specific neuronal populations and share common features of pathological deposition and aggregation of misfolded proteins [1, 2]. Alzheimer’s disease (AD), the most common NDD, is defined by amyloid-β (Aβ) plaques and neurofibrillary tangles composed of hyperphosphorylated tau [3]. Parkinson’s disease (PD), the fastest-rising NDD, features dopaminergic neuron degeneration in the substantia nigra pars compacta and Lewy bodies enriched with α-synuclein (α-Syn) [4]. Despite decades of research, the precise pathogenic mechanisms that initiate these disorders remain incompletely understood [5].

Accumulating epidemiological evidence has revealed an association between metabolic disorders and NDDs. Type 2 diabetes mellitus (T2DM) significantly elevates the risk of AD: patients with T2DM exhibit an approximately 50% increased risk of AD and a 70% higher risk of all-cause dementia [6–8]. Large-scale meta-analyses confirmed that T2DM increases dementia risk by 1.25–1.91 folds [9], and even prediabetes confers elevated risk of dementia [10]. Although the epidemiological evidence linking PD and T2DM remains less extensive than that for AD, existing data indicate that T2DM increases the subsequent risk of PD [11–13] and is associated with faster motor and cognitive decline in PD patients [14]. However, the mechanism underlying the comorbidity between T2DM and NDDs is incompletely defined. While shared pathogenic pathways have been proposed [15], a central question remains: how does a peripheral metabolic disorder affect central neurodegenerative processes?

A growing body of evidence points to brain insulin resistance (BIR) as a critical mechanistic link between the systemic metabolism dysfunction and central neurodegeneration [16, 17]. Insulin receptors (InSR) are widely expressed across different cell types in the central nervous system (CNS), including neurons, astrocytes, microglia, and oligodendrocytes [18]. Under physiological conditions, insulin signaling regulates not only cerebral glucose metabolism but also synaptic plasticity, neurotrophic support, neuroinflammation, and protein homeostasis [19, 20]. When insulin signaling becomes impaired, BIR occurs either through systemic metabolic dysfunction or through brain-intrinsic mechanisms, leading to dysregulation of these fundamental processes, setting the stage for neuronal deterioration [21, 22]. Moreover, emerging evidence indicates that BIR directly interferes with the protein pathology that defines AD and PD, such as Aβ, tau, and α-Syn [23]. This positions BIR as a potential upstream driver of proteinopathies among NDDs. Notably, BIR can occur independently of systemic insulin resistance and directly impairs cerebral energy homeostasis, synaptic function, and cell survival [24]. Therefore, elucidating how BIR contributes to protein aggregation and neurodegeneration represents a critical opportunity for identifying novel therapeutic targets and for interrupting the pathogenic cascade from early metabolic disorders to irreversible neuronal loss [20].

The present review has three primary aims. First, we examine how BIR disrupts the core functions of neurons, astrocytes, microglia, and oligodendrocytes, including energy metabolism, synaptic regulation and immunometabolic regulation. Second, we detail the recent findings on the underlying association between BIR and the pathological developments of Aβ deposition, tau hyperphosphorylation, and α-Syn aggregation. Finally, we critically evaluate existing therapeutic strategies targeting BIR, such as intranasal insulin administration and glucagon-like peptide-1 (GLP-1) receptor agonists, and discuss the translational challenges that have limited their clinical application. By integrating mechanistic insights with emerging therapeutic evidence, this review seeks to elucidate the molecular link underlying T2DM–NDDs comorbidity and to propose strategies that interrupt the transition from systemic metabolic dysfunction to BIR and subsequent neurodegeneration.

## BIR: cellular basis and cell-type specificity

**Fig. 1:**
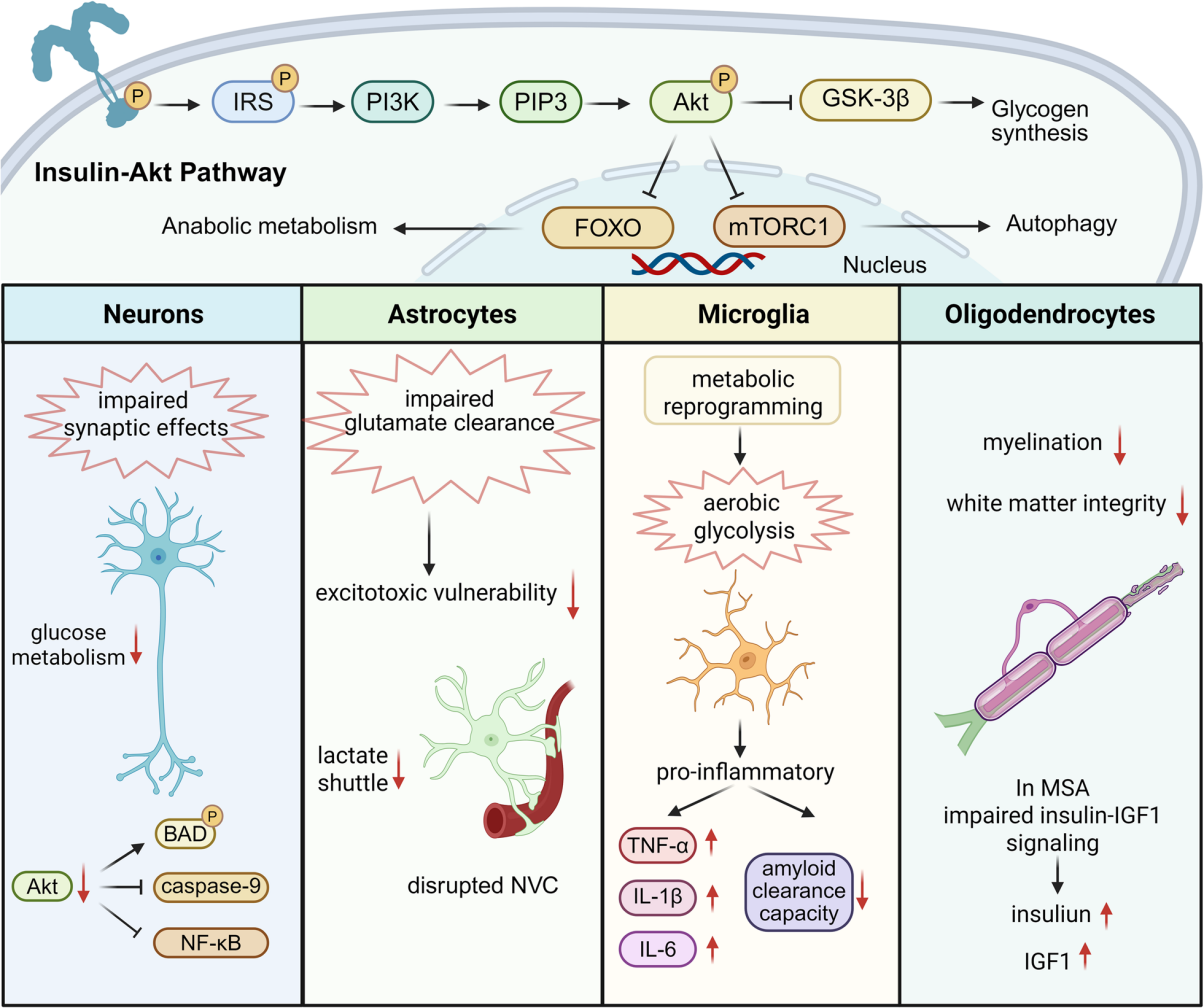
Cell-type-specific pathologies driven by brain insulin resistance. Top, the canonical Insulin-Akt signaling cascade. Insulin receptor activation induces IRS phosphorylation, triggering PI3K-mediated PIP3 generation and subsequent Akt activation. Activated Akt modulates downstream effectors including GSK-3β (promoting glycogen synthesis), FOXO (regulating anabolic metabolism), and mTORC1 (controlling autophagy). Bottom four panels, diverse cellular consequences of impaired insulin signaling across major CNS cell types. Neurons exhibit compromised synaptic function and reduced glucose metabolism; decreased Akt activity promotes BAD-mediated signaling, caspase-9 activation, and NF-κB pathway engagement. Astrocytes display impaired glutamate clearance and diminished lactate shuttle, disrupting neurovascular coupling (NVC) and increasing excitotoxic vulnerability. Microglia undergo metabolic reprogramming toward aerobic glycolysis, acquiring a pro-inflammatory phenotype characterized by elevated TNF-α, IL-1β, and IL-6 secretion alongside reduced amyloid clearance capacity. Oligodendrocytes show decreased myelination and compromised white matter integrity

BIR refers to a condition in which insulin signaling is impaired in cells of the CNS. BIR occurs independently of peripheral metabolic dysfunction or in conjunction with it, even in the absence of diabetes [25]. Under physiological conditions, insulin binding to InSR triggers autophosphorylation of the receptor β-subunit, which then phosphorylates insulin receptor substrate (IRS) proteins. Phosphorylated IRS recruits and activates phosphatidylinositol 3-kinase (PI3K), leading to the generation of PIP3 (phosphatidylinositol (3,4,5)-trisphosphate) and subsequent activation of serine/threonine kinase (Akt). Akt then phosphorylates multiple downstream targets, including glycogen synthase kinase-3β (GSK-3β), which is inhibited by this phosphorylation, FOXO transcription factors (excluded from the nucleus), and the mechanistic target of rapamycin complex 1 (mTORC1), which promotes anabolic metabolism and suppresses autophagy [26–28]. Importantly, insulin signaling is not uniform across neural cell types. Neurons, astrocytes, microglia, and oligodendrocytes each express the canonical InsR–IRS–PI3K–Akt cascade, but diverge substantially in their downstream effector engagement and functional outputs [29]. This cell-type specificity has a critical pathological corollary: when insulin signaling fails, each cell type deteriorates along its own functional trajectory, producing distinct but interacting contributions to the neurodegenerative milieu. The following sections discuss how physiological insulin signaling is organized in each cell type and how its disruption under BIR contributes to neuronal dysfunction, glial pathology, and ultimately proteinopathy (Fig. 1).

### Neurons: metabolic regulation, synaptic plasticity, and survival signaling

Neuronal insulin signaling directly regulates energy metabolism, glucose utilization, and bioenergetic support. Under physiological conditions, InSR activation stimulates glucose uptake via glucose transporters (GLUT) 4 translocation to enhance glycolytic flux in rat hippocampus and induces GLUT3 translocation in primary rat hippocampal cells [30, 31]. Positron emission tomography (PET) studies in humans have demonstrated reduced cerebral glucose metabolism in brain regions with high insulin receptor density in individuals with insulin resistance [32, 33]. This metabolic deficit results directly from the failure of insulin-stimulated glucose uptake under BIR. In addition to metabolism, insulin critically modulates synaptic function and cognitive performance. Insulin enhances NMDA receptor trafficking, promotes long-term potentiation through PI3K-Akt-mediated AMPA receptor insertion, and supports dendritic spine maintenance in mouse models [34, 35]. These synaptic effects are impaired under BIR condition, directly contributing to cognitive deficits in both AD and PD dementia [36, 37]. Furthermore, insulin signaling provides neuroprotection through survival pathways. Akt-mediated phosphorylation of BAD (Bcl-2-associated agonist of death), inhibition of caspase-9, and modulation of NF-κB protect against apoptotic and inflammatory insults in endothelial cells and rat cortical neurons [38–40]. This protective tone is attenuated in BIR states, increasing the vulnerability to secondary insults such as oxidative stress and protein aggregation, as shown in rats and SH-SY5Y cells [41, 42].

The neuronal specificity of insulin signaling leads to regional vulnerability to NDDs. Hippocampal CA1 neurons, critical for memory consolidation, exhibit high InSR expression and insulin-dependent glucose utilization [43]. Their susceptibility in early AD may partly reflect this metabolic dependence [44, 45]. Dopaminergic neurons of the substantia nigra pars compacta have lower InSR density, but are extraordinarily metabolically active due to their pacemaking activity and dopamine synthesis, making them sensitive to mitochondrial dysfunction consequent to BIR [46–48]. Cortical pyramidal neurons show progressive impairment of insulin signaling, correlating with Braak stage in postmortem AD brains, suggesting that BIR may propagate along neuroanatomical pathways in parallel with proteinopathy spread [49].

### Astrocytes: metabolic support, glutamate homeostasis, and neurovascular coupling

Astrocytes express InSR, and insulin signaling in these cells regulates metabolic support for neurons. Unlike neurons, astrocytes rely primarily on GLUT1 rather than GLUT4 for glucose uptake [50]. Insulin signaling enhances astrocytic glycogenolysis and lactate export through monocarboxylate transporters MCT1/2, supporting the astrocyte–neuron lactate shuttle that fuels neuronal activity in hGFAP-transgenic mice [51, 52]. Under BIR condition, this metabolic support is compromised. As shown in a familial AD model, reduced lactate availability forces neurons toward greater glucose self-utilization and compromises the astrocyte-derived metabolic buffering capacity required during periods of high neuronal activity [53].

Astrocytic insulin signaling also regulates glutamate homeostasis, protecting against excitotoxicity. Insulin promotes the expression of GLT-1 (glutamate transporter 1) and EAAT2 (excitatory amino acid transporter 2), which clear synaptic glutamate [54]. BIR impairs glutamate clearance, contributing to excitotoxicity, as shown in AβPP/PS1 mice and 6-hydroxydopamine-induced PD models [55, 56].

Neurovascular coupling, a process by which astrocytes translate neuronal activity into hemodynamic responses, is critically modulated by insulin signaling [57, 58]. Mice with astrocyte-specific knockout of insulin receptor exhibit a dissociation between cerebral blood flow and glucose uptake, demonstrating that BIR disrupts neurovascular coupling [59]. Accordingly, arterial spin labeling combined with resting-state functional MRI revealed AD-like neurovascular coupling alterations in patients with T2DM, characterized by decoupling between perfusion and brain functional connectivity at rest within the default mode networks [60]. The relative contribution of astrocytic versus neuronal insulin resistance to the overall disease pathogenesis remains incompletely defined, and therapeutic strategies specifically targeting astrocytes have not yet been developed [61, 62].

### Microglia: immunometabolic switching and phagocytosis

Microglial insulin signaling is a critical regulator of immunometabolic phenotype, determining whether microglia adopt a protective or a destructive state [63, 64]. InSR activation promotes microglial transition toward an anti-inflammatory, phagocytically competent state characterized by enhanced Aβ uptake and degradation, increased expression of phagocytic receptors including TREM2 (triggering receptor expressed on myeloid cells 2) and CD36, and reduced production of pro-inflammatory cytokines [65, 66]. Conversely, BIR drives microglial polarization toward a pro-inflammatory, phagocytically impaired state with elevated secretion of tumor necrosis factor-α, interleukin (IL)-1β, and IL-6 and reduced amyloid clearance capacity, as shown in mice with microglia-specific insulin receptor knockout [67]. This immunometabolic reprogramming is mediated mainly through the PI3K–Akt signaling pathway. BIR shifts microglial glucose metabolism from oxidative phosphorylation toward aerobic glycolysis, a metabolic mode associated with inflammatory activation [68, 69].

### Oligodendrocytes: myelination and white matter integrity

Insulin signaling promotes oligodendrocytic precursor cell proliferation, differentiation, and myelination through InSR [70]. In patients with multiple system atrophy, the insulin–IGF1 signaling is impaired in both neurons and oligodendrocytes, accompanied by compensatory increases in plasma insulin and IGF1 [70, 71]. BIR has been associated with white matter abnormalities in both AD and PD patients, but direct mechanistic links to amyloid proteinopathy in white matter remain less established. White matter changes may reflect secondary consequences of neuronal and axonal degeneration rather than primary oligodendrocyte pathology [72].

## BIR regulates amyloid protein pathology

**Fig. 2:**
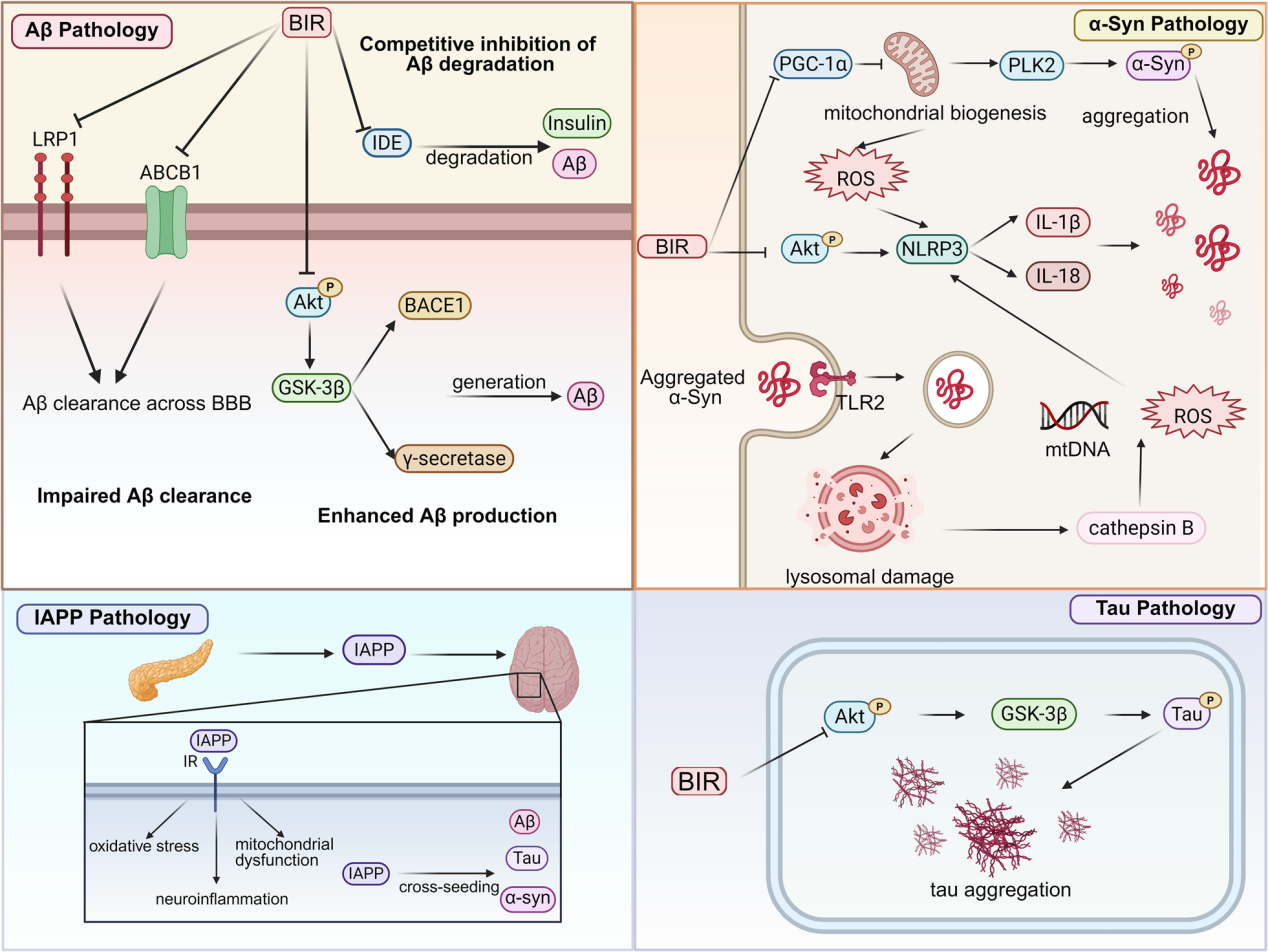
Brain insulin resistance drives multiple proteinopathies. BIR impairs Aβ clearance across the blood–brain barrier by downregulating LRP1 and ABCB1, and competitively inhibits IDE-mediated Aβ degradation. Suppressed Akt activity downstream of BIR activates GSK-3β, which promotes Aβ production via BACE1 and γ-secretase and drives tau hyperphosphorylation and aggregation. In α-Syn pathology, BIR inhibits the PGC-1α/PLK2 axis, aggravating α-Syn phosphorylation and aggregation, and activates the NLRP3 inflammasome to induce IL-1β/IL-18 release and lysosomal damage. Furthermore, IAPP translocates to the brain and, acting through the InSR, elicits oxidative stress, mitochondrial dysfunction, and neuroinflammation. IAPP cross-seeds with Aβ, tau, and α-Syn, accelerating pathological aggregation across these substrates

The cell-type-specific disruptions caused by BIR do not merely compromise neuronal or glial function, but rather converge on a common downstream consequence: the failure of proteostasis. In this section, we examine how BIR directly dysregulates the metabolism of Aβ, tau and α-Syn, three amyloid proteins that define AD and PD. BIR engages each through distinct molecular pathways, leading to common consequences including oxidative stress, impaired autophagy, and chronic inflammation. Beyond these brain-intrinsic mechanisms, systemic insulin resistance generates a peripherally derived amyloidogenic protein, islet amyloid polypeptide (IAPP), that cross-seeds existing aggregates, functioning as an extracerebral amplifier of the proteinopathy already initiated by BIR within the CNS (Fig. 2).

### BIR disrupts Aβ clearance and promotes its production

BIR dysregulates Aβ homeostasis through three convergent mechanisms: impairing blood–brain barrier (BBB) efflux, compromising the insulin-degrading enzyme (IDE)-mediated Aβ degradation, and enhancing the GSK-3β-driven production of Aβ.

First, BIR inhibits Aβ clearance across the BBB by downregulating key transporters. Intact insulin signaling maintains the functions of low-density lipoprotein receptor related protein 1 (LRP1) and ABCB1 (ATP-binding cassette subfamily B member 1, also named P-glycoprotein), which actively transport Aβ from brain parenchyma to peripheral circulation [73–76]. Knockdown of LRP1 in primary astrocytes or its genetic deletion in APP/PS1 mice reduces Aβ efflux and accelerates plaque deposition [77]. Brain endothelial cell-specific loss of LRP1 leads to a marked decrease in Aβ efflux from the brain [74]. Similarly, deficiency of P-glycoprotein results in significantly elevated Aβ accumulation in mice following intravenous injection of [^125^I]-labeled Aβ [78]. These transporters are expressed in both endothelial cells and astrocytes, suggesting the importance of intact insulin signaling in multiple cell types for BBB efflux. However, these findings were obtained primarily from gene knockout models, in which the phenotypic severity likely exceeds the degree of functional downregulation occurring under physiological BIR [79, 80].

Second, BIR causes substrate competition for IDE, which is a canonical molecular node linking hyperinsulinemia to impaired Aβ clearance. IDE degrades both insulin and Aβ, but exhibits markedly higher affinity to insulin [81]. ^1^⁸O-isotope labeling mass spectrometry in human cerebrospinal fluid (CSF) has validated this dual degradative capacity [82]. Notably, under physiological conditions, IDE expression is regulated by insulin signaling pathways, whereby InSR activation promotes IDE expression in astrocytes through ERK-dependent signaling, thereby enhancing Aβ degradation [83]. However, in hyperinsulinemia resulting from systemic insulin resistance, the increased insulin competitively inhibits Aβ degradation. In AD patient cortex and transgenic mouse models, IDE expression is upregulated likely as a compensatory response, but this adaptation is negated by substrate competition [84]. Therefore, insulin exerts a context-dependent dual effect on Aβ metabolism: physiological insulin signaling maintains IDE expression and facilitates Aβ clearance, whereas pathological insulin resistance and hyperinsulinemia impair IDE function and contribute to Aβ accumulation. These findings suggest that simply increasing insulin levels may not represent an effective therapeutic strategy for enhancing Aβ clearance..

Last but not least, studies in APP23/PS45 double transgenic mice and neuronal cell lines reported that BIR reduces Akt activity, leading to GSK-3β disinhibition. The enhanced GSK-3β activity upregulates β-secretase (BACE1) and γ-secretase, shifting APP processing toward Aβ generation while inhibiting the non-amyloidogenic α-secretase pathway [85, 86]. However, this mechanistic framework has yet to be confirmed in human brain tissues [87]. Moreover, GSK-3β has over 100 known substrates, and its activation can be driven by Wnt antagonism, oxidative stress, or inflammatory mediators independent of BIR. This makes it a pharmacologically non-specific node, which should be critically considered when evaluating GSK-3β inhibitors as therapeutic candidates.

### BIR exacerbates tau hyperphosphorylation

GSK-3β is the major tau kinase, and its disinhibition in BIR drives tau hyperphosphorylation at multiple pathological epitopes. In BIR states, reduction of Akt-mediated phosphorylation of GSK-3β (Ser9) relieves inhibitory control, enhancing tau phosphorylation [88, 89]. In transgenic *Drosophila* expressing human tau and in insulin-treated SH-SY5Y neuroblastoma cells, insulin resistance increases tau phosphorylation at AT8 and PHF1 epitopes and promotes tau aggregation through the Akt–GSK-3β signaling axis [90]. Further studies are needed to clarify whether reduction of PP2A-mediated tau dephosphorylation and impaired autophagic clearance of tau oligomers, which are cellular events closely related to amyloid protein accumulation, also contribute to tau phosphorylation.

More broadly, the causal relationship between BIR and tau pathology in human AD, while strongly supported by preclinical data, has not been demonstrated at the level of direct causal evidence. Postmortem AD brains show elevated GSK-3β activity alongside tau hyperphosphorylation [91, 92], but cross-sectional analyses could not determine the causal effects. A landmark study by Talbot and colleagues identified progressive increases in IRS-1 serine phosphorylation in MCI and AD patient brains, correlating with memory deficits and oligomeric Aβ plaques. However, this study did not measure GSK-3β activity or site-specific tau phosphorylation, leaving the complete BIR-Akt-GSK-3β-tau causal axis unsubstantiated in human tissue [93].

### BIR modulates α-Syn aggregation and phosphorylation

BIR promotes α-Syn aggregation through two pathways: the direct Polo-like kinase 2 (PLK2)-mediated phosphorylation pathway, and the indirect pathway via neuroinflammation and NOD-like receptor family pyrin domain containing 3 (NLRP3) inflammasome activation. PLK2 is a key kinase linking BIR-induced mitochondrial impairment to α-Syn phosphorylation. BIR impairs mitochondrial biogenesis via peroxisome proliferator-activated receptor γ coactivator 1-α (PGC-1α) suppression, leading to PLK2 upregulation, which modulates α-Syn phosphorylation. In high-fat diet MitoPark diabetic mice and human differentiated dopaminergic neurons, insulin resistance suppresses PGC-1α, increasing oxidative stress and PLK2 activity. PLK2 modulates α-Syn phosphorylation at Ser129 [94, 95]. PLK2 inhibition reverses insulin resistance-induced elevations in the total and the phosphorylated α-Syn [96].

The second pathway linking BIR to α-Syn pathology is through microglial inflammation. As described above, BIR drives a pro-inflammatory state of microglia. A key mechanism is the loss of insulin-mediated restraint on the NLRP3 inflammasome. Under normal conditions, Akt phosphorylates NLRP3 to prevent its oligomerization, and insulin suppresses ASC assembly and the NF-κB-driven NLRP3 expression [97]. In BIR condition, the failure of these inhibitory mechanisms result in NLRP3 activation, driving IL-1β and IL-18 release. These cytokines exacerbate α-Syn pathology through multiple mechanisms, including promoting oxidative stress, impairing autophagic clearance, and directly enhancing α-Syn phosphorylation [98]. Crucially, aggregated α-Syn is itself a potent NLRP3 activator, recognized by TLR2 on microglia and capable of triggering the same inflammasome cascade [98]. This establishes a self-amplifying cycle: BIR disinhibits NLRP3, the resulting inflammation worsens α-Syn pathology, and aggregated α-Syn further activates NLRP3. This bidirectional relationship makes it difficult to establish causal direction between BIR and human PD. Unlike AD, where Aβ and tau pathologies have relatively well-characterized temporal relationships, PD has a prodromal phase extending over two decades [99, 100], during which BIR and α-Syn pathology may reinforce each other in ways that cross-sectional studies cannot disentangle.

### IAPP deposition and cross-seeding: a peripheral amplifier of central proteinopathy

The mechanisms described above focus on how BIR within the CNS drives proteinopathy. But systemic insulin resistance also generates a peripherally derived amyloidogenic protein, IAPP. IAPP enters the brain and directly amplifies the aggregation of Aβ, tau, and α-Syn.

IAPP is co-secreted with insulin from pancreatic β-cells. Under insulin-resistant conditions, the compensatory hypersecretion elevates IAPP, promoting downstream cross-seeding with NDD-related pathological proteins [101, 102]. Although IAPP mRNA is not detected in the brain, IAPP aggregates are found in AD- and PD-associated brain regions, indicating peripheral origin with subsequent BBB transit [103, 104]. In the CNS, IAPP engages amylin receptors to trigger oxidative stress, neuroinflammation, and mitochondrial dysfunction, exerting toxicity independently of its cross-seeding activity.

The cross-seeding capacity of IAPP arises from its structural compatibility with multiple amyloidogenic proteins. IAPP shares approximately 25% sequence identity with Aβ, and the Aβ(24–34) and IAPP(19–29) segments adopt nearly identical backbone conformations. The residues F23 and I26 in human IAPP mediate both self-aggregation and cross-seeding with Aβ [105, 106]. With respect to tau, IAPP directly binds the R3 fragment of tau, inducing a β-hairpin conformation that promotes tau oligomerization [107, 108]. IAPP also accelerates α-Syn aggregation through interactions via its C-terminal residues [109, 110].

The evidence for the role of IAPP arose from multiple experimental systems. Postmortem analyses have revealed co-deposition of IAPP with pathogenic proteins in disease-relevant regions. In AD patients, co-deposits are found in the temporal cortex, hippocampus, and pancreatic islets. In PD subjects, they are detected in pancreatic islets and the substantia nigra [103, 111–115]. Single-transgenic mouse studies confirmed that IAPP promotes Aβ aggregation and exacerbates memory deficits, while in tau pathology models it enhances phosphorylation, seeding activity, and synaptic dysfunction [103, 116]. In spontaneously diabetic cynomolgus monkeys, a model that recapitulates the spontaneous metabolic deterioration of human T2DM, α-Syn and IAPP colocalize in pancreatic islets and brain, with synchronous α-Syn accumulation and IAPP upregulation [117, 118]. Dual-transgenic models expressing hAβ/hIAPP and hTau/hIAPP reveal synergistic effects on both metabolic and neurodegenerative endpoints [119, 120]. Collectively, evidence from patient tissues to transgenic rodents to non-human primates, establishes IAPP as a molecular bridge linking peripheral insulin resistance to central proteinopathy.

However, a notable gap is that existing studies have focused almost exclusively on unidirectional effects, examining how peripheral IAPP seeds cerebral pathology. Whether brain-derived Aβ or tau can, in turn, accelerate pancreatic IAPP aggregation, thereby forming a vicious cycle between the brain and the pancreas, remains underexplored and represents an important direction for future investigation.

## Therapeutic strategies targeting the BIR–neurodegeneration axis

Having established how BIR drives both cell-type-specific disfunction and the pathological accumulation of Aβ, tau, and α-Syn, we now evaluate therapeutic strategies that target one or both mechanisms. We then examine the clinical evidence, revealing that human trials consistently have yielded at best modest and often negative results, despite compelling preclinical data. For each therapeutic strategy, we therefore ask what this disconnect tells us about the nature of BIR-driven neurodegeneration and the limitations of current therapeutic paradigms. We propose the identification of structural reasons for repeated translational failure and propose a path toward multi-target strategies that engage the full complexity of BIR pathology (Table 1**)**.

**Table 1:** Anti-diabetic agents in the treatment of neurodegenerative diseases

	Intranasal insulin	Metformin	TZDs	GLP-1 receptor agonists
Representative agents	Regular insulin (Novolin R, Humulin R) Insulin detemir (Levemir)	Metformin HCl (Glucophage, Fortamet, Glumetza, Riomet) Metformin ER	Pioglitazone (Actos) Rosiglitazone (Avandia)	Liraglutide (Victoza) Semaglutide (Ozempic) Dulaglutide (Trulicity) Exenatide (Byetta)
Onset of action	Rapid (minutes to hours [123])	Slow (weeks to months [157])	Moderate (days to weeks) [198]	Moderate (days to weeks) [187]
Duration of action	Short (multiple daily doses) [127]	Long (once daily)	Long (1–2 times daily)	Long (daily to weekly)
Certainty of CNS exposure	Low (olfactory bulb targeting, uneven brain distribution) [123]	Moderate (partial BBB penetration)	Low (limited brain concentrations) [198]	Moderate (partial BBB penetration + gut-brain axis [173])
Functional targets	Insulin receptors IGF-1 receptors PI3K/Akt/GSK-3β [128] IDE (enhanced Aβ degradation) [129]	AMPK [143] PI3K/Akt [144] Mitochondrial complex I [199] NF-κB pathway [200, 201] mTOR and autophagy [147] Aβ precursor protein levels [144, 145]	PPARγ C1q (Aβ clearance) [160] Inflammatory cytokines [202] (TNF-α, IL-6 reduction)	GLP-1R Wnt/β-catenin [174, 175] PI3K/Akt/GSK-3β [203] BACE1 [176] SIRT1-Glut4 [178]
Clinical trial	Meta-analysis (*n* = 8) deemed insufficient evidence for efficacy [134] 12-month randomized controlled trial showed no benefits in cognition/function [135] Pilot studies show cognitive benefits [204]	Reduces the risk of dementia and delirium [148, 149] Randomized controlled trial: 40-week treatment improved brain glucose uptake and preserved volume [150]	A retrospective cohort analysis showed that pioglitazone reduces dementia risk in diabetes [164] A phase 3 trial (TOMORROW, pioglitazone) demonstrated no efficacy [165]	Multiple cohort studies and a phase 2b clinical trial suggest reduced AD and PD incidence [184–187]
Major limitations	Risk of receptor desensitization	GI side effects [157]	Systemic safety risks [168]	GI side-effects; injection burden [187]
Optimal target population	Early AD, *APOE*-ε4 non-carriers [137]	Patients with comorbid metabolic syndrome	Prediabetes, obesity, economically constrained	Early PD, comorbid GI dysfunction

Aβ, amyloid-beta; AD, Alzheimer’s disease; AMPK, AMP-activated protein kinase; APOE-ε4, apolipoprotein E epsilon 4; BACE1, β-site amyloid precursor protein cleaving enzyme 1; BBB, blood–brain barrier; C1q, complement component 1q; CNS, central nervous system; ER, extended release; GI, gastrointestinal; GLP-1, glucagon-like peptide-1; GLP-1R, glucagon-like peptide-1 receptor; GLUT4, glucose transporter 4; GSK-3β, glycogen synthase kinase-3 beta; HCl, hydrochloride; IDE, insulin-degrading enzyme; IGF-1, insulin-like growth factor-1; IL-6, interleukin-6; mTOR, mechanistic target of rapamycin; NF-κB, nuclear factor kappa B; PD, Parkinson’s disease; PI3K, phosphatidylinositol 3-kinase; PPARγ, peroxisome proliferator-activated receptor gamma; SIRT1, sirtuin 1; TNF-α, tumor necrosis factor alpha; TZDs, thiazolidinediones; Wnt, wingless-related integration site

### Intranasal insulin: direct ligand supplementation

Intranasal insulin administration uses the olfactory and trigeminal nerve pathways to deliver insulin directly to the CNS, bypassing the BBB and achieving therapeutically relevant concentrations in the CSF, olfactory bulb, hippocampus, and cortex [121–123]. Because this route causes only marginal elevations in peripheral serum insulin, the risk of systemic hypoglycemia is minimal [124–127]. The mechanistic rationale for this approach is straightforward: if BIR reflects insufficient insulin signaling, then supplementing the ligand should restore the downstream pathway activity. Indeed, intranasal insulin engages the canonical PI3K–Akt cascade, upregulates the anti-apoptotic protein Bcl-2, suppresses the oxidative stress-induced caspase-3 activation and DNA fragmentation, and reverses the aberrant upregulation of glutathione peroxidase-1 in primary cortical neurons [40, 128, 129]. These effects should, in principle, rescue both the neuronal survival deficits and the proteostasis, promoting IDE-mediated Aβ degradation, LRP1-dependent Aβ efflux across the BBB, and GSK-3β inhibition to reduce tau hyperphosphorylation. Such beneficial outcomes have been demonstrated in various transgenic mouse models, including APP/PS1 and 3 × Tg-AD mice, as well as in rat models [130–133].

Clinical evidence, however, has not fulfilled this promise. A systematic review and meta-analysis that screened 647 articles and ultimately included 8 studies found substantial heterogeneity across trials and concluded that the evidence remains insufficient to establish intranasal insulin as a routine treatment for AD [134]. A randomized clinical trial of 12-month intranasal insulin therapy reported no cognitive or functional benefits over placebo [135]. Several factors may account for this variability. First, the preferential distribution of intranasally delivered insulin to the olfactory bulb may result in heterogeneous brain exposure, with inadequate delivery to deeper structures such as the hippocampus and substantia nigra [136]. Second, the inter-individual differences in nasal anatomy and mucosal integrity, and the absence of standardized delivery devices contribute to substantial pharmacokinetic variability [136]. Third, the treatment response is significantly modulated by the *APOE* genotype, with ε4 non-carriers receiving greater benefits, whereas ε4 carriers showing no improvement or even worsening of disease [137].

These findings illustrate a broader problem: supplementing the ligand assumes that the downstream drug-targeting and signaling machinery is intact and capable of responding. First, achieving reliable, region-specific CNS insulin exposure remains an unsolved challenge, compounded by interindividual anatomical variability and the absence of standardized devices [136, 138, 139]. Second, even when insulin reaches the brain, its efficacy depends on factors in the host, most notably *APOE* genotype, which is rarely used to stratify trial participants [137]. These problems create a fundamental ambiguity: we cannot determine if the clinical trial failure could be attributed to inadequate engagement of the drug target, or to the disruption of the insulin signaling cascade that failed to respond to ligand supplementation. At minimum, imaging-based confirmation of regional brain distribution, standardized delivery protocols, and mandatory stratification by patient genotypes are needed in future trials to resolve this ambiguity.

### Metformin: insulin sensitization beyond the receptor

Metformin is an insulin sensitizer that enhances the cellular response to endogenous insulin rather than supplementing exogenous ligand [140]. The primary mechanism involves AMPK activation, which improves insulin sensitivity, reduces oxidative stress, and suppresses neuroinflammation [141, 142]. AMPK activation by metformin enhances neuronal energy metabolism and supports survival signaling under conditions of BIR. At the level of protein pathology, metformin-mediated activation of the AMPK–ULK1 cascade ameliorates mitochondrial structural and functional deficits while reducing Aβ burden in 3 × Tg-AD and APP/PS1 mice [143–145]. Conversely, in MPTP-induced PD models, metformin promotes the AMPK-dependent autophagy to facilitate α-Syn clearance [146, 147]. Cohort studies have further suggested that long-term metformin use is associated with reduced dementia risk and lower rates of mild cognitive impairment [148, 149]. A recent randomized controlled trial demonstrated that 40 weeks of metformin treatment (2000 mg/day) in older adults with insulin resistance improved brain glucose uptake, strengthened the cognitive network connectivity, and preserved brain volumes [150].

However, the therapeutic window of metformin is narrow and its effects are context-dependent. AMPK overactivation can inhibit mTORC1 to a degree that impairs protein synthesis and synaptic plasticity [151–153], and prolonged AMPK activation may precipitate neuronal apoptosis under conditions of energy stress [154, 155]. Therefore, early and moderate metformin use may confer benefits, while late or excessive administration could be detrimental [156, 157]. Clinical evidence in PD remains particularly limited. Metformin thus exemplifies a fundamental challenge in targeting BIR: the same signaling node can be protective or harmful depending on the disease stage, the cell type, and the degree of pathway activation—a consideration that is rarely captured in standard clinical trial designs.

### Thiazolidinediones (TZDs): PPARγ-mediated transcriptional activation

TZDs, including rosiglitazone and pioglitazone, are selective ligands for the nuclear transcription factor peroxisome proliferator-activated receptor γ (PPARγ). PPARγ is functionally expressed in neurons, astrocytes, and microglia, making TZDs conceptually attractive as multi-cell-type interventions [158, 159]. In APP/PS1 mouse models, pioglitazone significantly attenuates the C1q-mediated synaptic tagging, inhibits the microglial phagocytic activity, and stabilizes synaptic density during early pathological stages [160]. At the proteinopathy level, pioglitazone upregulates IDE expression and inhibits BACE1 via PPARγ target genes, thereby promoting Aβ degradation while suppressing its production in primary rat hippocampal neurons [161]. In parallel, in a rat model of progressive α-synucleinopathy, pioglitazone reduces α-Syn aggregation, ameliorates behavioral deficits, and attenuates dopaminergic neuronal loss [162].

The clinical trajectory of TZDs, however, has been one of early promise followed by decisive failure. Pioglitazone treatment for 6 months in diabetic AD patients showed positive effects on cognition, neurometabolic function, and neuroinflammation [163, 164]. But the TOMORROW trial, a large phase 3 study, found that long-term low-dose pioglitazone (0.8 mg/day) did not delay the onset of mild cognitive impairment in cognitively normal older adults at high AD risk [165]. Similarly, early-phase trials (*n* < 100) of rosiglitazone suggested memory improvement and plasma Aβ_42_ stabilization, and a phase 2 study reported cognitive benefits in *APOE*-ε4 non-carriers. However, two large phase 3 trials confirmed no clinically significant efficacy across genotypes [166].

The failure of TZDs in treating NDDs fundamentally stems from the stark asymmetry in drug distribution between peripheral tissues and the CNS. These drugs penetrate the BBB poorly, resulting in markedly lower concentrations in the brain than in peripheral tissues, and this insufficient central exposure likely undermines their target engagement, preventing the neuroprotective effects observed in preclinical models from being realized [167]. Meanwhile, their high peripheral exposure may lead to unacceptable toxicity, which has severely restricted their clinical use. Rosiglitazone has been marginalized or withdrawn in most countries after meta-analyses suggested it increases the risk of myocardial infarction and cardiovascular death [168]. Pioglitazone, the only routinely available TZD globally, is associated with a clear time- and dose-dependent elevated risk of bladder cancer with long-term use [169]. The clinical application of pioglitazone now requires stringent patient selection—excluding individuals with a history of bladder cancer, heart failure, or those at a high risk of osteoporosis—and cautious use only after a thorough risk–benefit assessment [170]. The peripheral toxicity combined with insufficient central efficacy lies at the heart of the failure of clinical translation of TZDs. In addition, the failure of drugs to reach targets in the human CNS at adequate concentrations is an obstacle for translation despite their compelling preclinical pharmacological mechanisms.

### GLP-1 receptor agonists: receptor mimetic activation

GLP-1 receptor agonists represent the most promising class of BIR-targeting therapeutics currently under investigation. These agents can cross the BBB and enhance the central insulin signaling while exerting multimodal neuroprotective effects [171–173]. Their mechanisms engage both levels of pathology identified in this review. At the cellular level, exendin-4 promotes endogenous insulin production in the brain via Wnt-β–catenin–NeuroD1 signaling, boosting the intrinsic insulin-synthesizing capacity in the CNS rather than relying on exogenous replacement [174, 175]. Liraglutide specifically restores IRS-1 tyrosine phosphorylation and Akt activation through the IRS-1–Akt–GSK-3β pathway [176, 177], and semaglutide regulates GLUT4 expression via SIRT1, improving glucose metabolic dysfunction in AD models [178]. At the protein level, exendin-4 reduces the BACE1-mediated APP processing and Aβ accumulation in APP/PS1 mice [179]; liraglutide prevents hippocampal tau hyperphosphorylation in db/db mice [177]; and semaglutide reduces Aβ plaques and neurofibrillary tangles in 3 × Tg mice [178]. In PD models, exendin-4 alleviates α-Syn pathology through enhanced autophagy [180]. GLP-1 receptor agonists also engage a unique gut-brain axis mechanism: they upregulate intestinal barrier-protective molecules including claudin-7, IL-33, and Mucin 5b [181, 182], providing a basis for blocking the propagation of α-Syn pathology from the gut to the CNS. However, this is an emerging research area and more studies are needed to confirm this mechanism [183].

Clinically, the signals are encouraging but not yet definitive. Exenatide has shown modest improvements in motor function in PD patients, with larger confirmatory trials ongoing [184]. A Scandinavian cohort study found that GLP-1 receptor agonist use was associated with an approximately 19% lower incidence of PD compared with sulfonylurea use [185], and semaglutide and liraglutide have been associated with a significantly reduced risk of newly-onset AD in patients with T2DM [186]. However, these are observational and retrospective data; randomized controlled trials with neurodegenerative endpoints remain sparse. Moreover, a substantial proportion of patients experience pronounced gastrointestinal adverse effects and significant weight loss [187], which may limit the long-term tolerability in elderly NDDs populations.

GLP-1 receptor agonists are, in many aspects, the most mechanistically comprehensive BIR-targeting strategy currently available: they enhance insulin signaling, engage both sides of the dual mechanism identified in this review, and address the gut-brain dimension of PD pathology. Yet, the gap between epidemiological suggestion and randomized evidence remains wide. Trials that are explicitly designed for neurodegenerative endpoints and incorporate patient stratification factors including *APOE* genotype, sex, diabetes comorbidity, and disease stage, are critical for bridging this gap.

### Post-receptor strategies: targeting downstream effectors

Post-receptor strategies refer to therapeutic approaches that target intracellular signaling molecules downstream of the InSR, aiming to restore or modulate impaired insulin signaling. Several strategies, including IRS serine phosphorylation inhibitors, GSK-3β inhibitors, and FOXO modulators, aim to modulate the intracellular effectors of insulin signaling rather than the receptor itself [188]. The rationale is appealing: if BIR impairs signaling at or downstream of IRS-1, then targeting these nodes could bypass the receptor-level defects.

Compelling preclinical evidence supports this logic. In NLRP3 knockout mice, Aβ_1-42_ injection failed to increase IRS-1 serine phosphorylation, and intact insulin signaling was maintained despite Aβ exposure, suggesting that targeting the upstream inflammatory drivers of IRS-1 phosphorylation may restore pathway function more effectively than direct insulin replacement [189]. The novel GSK-3β inhibitor ING-135 ameliorates cognitive decline and reduces tau phosphorylation with no overt toxicity in the hTau/PS1 model [190]. FOXO proteins, given their role in oxidative stress responses and longevity pathways, are also regarded as potential targets for BIR and AD [191].

However, the translational distance for these strategies is greater than for any of the drug classes discussed above. No IRS-1 phosphorylation inhibitor has entered clinical trials for AD or PD, a reflection of both the technical difficulty of achieving substrate-specific kinase inhibition and the theoretical risk of excessive pathway activation. Furthermore, the downstream effectors of Akt exhibit what might be called a hierarchical sensitivity to reduced insulin signaling. In mild BIR, GSK-3β is already disinhibited, whereas mTORC1 activity remains sufficient to suppress autophagy; FOXO nuclear translocation typically requires more severe or sustained signaling impairment [25, 192, 193]. This implies that the therapeutic window for each downstream target shifts with disease stage: early BIR may respond to GSK-3β inhibition, whereas advanced BIR may require autophagy restoration or FOXO modulation. Developing biomarkers for the activation state of specific pathway nodes, such as IRS-1 phosphorylation status in CSF or extracellular vesicles, that enables patient stratification, may therefore be a prerequisite for the clinical translation of post-receptor strategies.

### A critical reappraisal: why have BIR-targeting therapies not yet succeeded?

Collectively, the clinical experience reviewed above, spanning intranasal insulin, metformin, TZDs, GLP-1 receptor agonists, and post-receptor strategies, reveals a consistent translational gap across drug classes. Preclinical studies robustly demonstrated that restoring insulin signaling can mitigate both the cellular dysfunction described and the proteinopathy. Yet in human trials, the effects are at best modest and often absent.

This disconnect, we argue, reflects a fundamental mismatch between the complexity of BIR-driven pathology and the simplicity of current therapeutic interventions. BIR is not a unitary condition but a composite of four distinct cell-type-specific derangements, including synaptic failure in neurons, metabolic uncoupling in astrocytes, immunometabolic reprogramming in microglia, and impaired oligodendrocyte myelination. Each derangement interacts and amplifies one another. Simultaneously, BIR directly dysregulates the metabolism of Aβ, tau, and α-Syn through multiple converging pathways, while peripheral IAPP cross-seeding adds another layer of proteotoxic stress.

Emerging combination strategies have begun to show promise in overcoming this complexity where single agents have fallen short. For instance, intranasal insulin combined with the GLP-1 receptor agonist semaglutide has been shown to improve the cognitive performance in elderly individuals with mild cognitive impairment [194]. Additionally, novel dual GLP-1/gastric inhibitory polypeptide (GIP) receptor agonists demonstrate superior neuroprotective efficacy compared with single GLP-1 or GIP receptor agonists alone [195, 196]. Enhanced BBB penetration appears to be a critical factor underlying this improved therapeutic outcome [197]—a finding that directly echoes the lessons learned from the clinical translation of TZDs, which failed due to insufficient central exposure.

These pioneering studies collectively suggest that the core strategy must be the integration of insulin signaling enhancement with restoration of proteostasis. We therefore propose rationally designed multi-target strategies for the next-generation BIR-targeting therapies: combining agents to simultaneously restore cell-type-specific insulin signaling, enhance proteostatic clearance, and block cross-seeding interactions, in patient populations stratified by genotype, disease stage, and metabolic comorbidity. Without such a paradigm shift, the gap between preclinical promise and clinical failure will persist.

## Conclusion

BIR is not a single pathological entity. It is a composite of cell-type-specific derangements, including synaptic failure in neurons, metabolic uncoupling in astrocytes, immunometabolic reprogramming in microglia, and impaired oligodendrocyte myelination. These converge to drive the proteostatic collapse underlying AD and PD. This mechanistic complexity explains a pattern that has frustrated the field for over a decade: preclinical studies repeatedly demonstrate that restoring insulin signaling mitigates both cellular dysfunction and proteinopathy, yet clinical trials of intranasal insulin, metformin, TZDs, and GLP-1 receptor agonists have yielded at best modest and often negative results. The reason, we argue, is structural. A therapeutic strategy that addresses one node in a pathological network, whether by supplementing the ligand, sensitizing the receptor, or inhibiting a single downstream kinase, cannot reverse the full spectrum of BIR-driven neurodegeneration. The path forward lies in rationally designed multi-target strategies that simultaneously restore cell-type-specific insulin signaling, enhance proteostatic clearance, and block cross-seeding interactions, in patient populations stratified by genotype, disease stage, and metabolic comorbidity. Progress will also require CNS-optimized delivery systems and biomarkers that capture the activation state of specific insulin signaling nodes. The framework offered here, connecting cell-type-specific BIR pathology to proteostatic failure and therapeutic opportunity, is intended to move the field toward the design of interventions that match the complexity of the disease they aim to treat.

## Data Availability

No datasets were generated or analysed during the current study.

## Abbreviations

AD: Alzheimer’s disease
Akt: Akt serine-threonine protein kinase
Aβ: Amyloid-β
BACE1: β-Secretase
BBB: Blood–brain barrier
BIR: Brain insulin resistance
CNS: Central nervous system
CSF: Cerebrospinal fluid
GLP-1: Glucagon-like peptide-1
GIP: Gastric inhibitory polypeptide
GLUT: Glucose transporters
GSK-3β: Glycogen synthase kinase 3β
IAPP: Islet amyloid polypeptide
IDE: Insulin-degrading enzyme
IL: Interleukin
InSR: Insulin receptor
IRS: Insulin receptor substrate
LRP1: Lipoprotein receptor-related protein 1
mTORC1: Mechanistic target of rapamycin complex 1
NDDs: Neurodegenerative diseases
NLRP3: NOD-like receptor family pyrin domain containing 3
PD: Parkinson’s disease
PET: Positron emission tomography
PGC-1α: Peroxisome proliferator-activated receptor γ coactivator 1-α
PI3K: Phosphatidylinositol 3-kinase
PLK2: Pathways involving polo-like kinase 2
PPARγ: Peroxisome proliferator-activated receptor γ
T2DM: Type 2 Diabetes mellitus
TZDs: Thiazolidinediones
α-Syn: α-Synuclein

## References

[1] Wilson DM 3rd, Cookson MR, Van Den Bosch L, Zetterberg H, Holtzman DM, Dewachter I. Hallmarks of neurodegenerative diseases. Cell. 2023;186(4):693–714. 36803602 10.1016/j.cell.2022.12.032

[2] Dugger BN, Dickson DW. Pathology of neurodegenerative disease. Cold Spring Harb Perspect Biol. 2017;9(7):a28035. 10.1101/cshperspect.a028035 PMC5495060 28062563

[3] Mucke L. Neuroscience: Alzheimer’s disease. Nature. 2009;461(7266):895–7. 19829367 10.1038/461895a

[4] Kalia LV, Lang AE. Parkinson’s disease. Lancet. 2015;386(9996):896–912. 25904081 10.1016/S0140-6736(14)61393-3

[5] Lau VZ, Awogbindin IO, Frenkel D, Whitehead SN, Tremblay M. A hypothesis explaining Alzheimer’s disease, Parkinson’s disease, and dementia with Lewy bodies overlap. Alzheimers Dement. 2025;21(6):e70363. 40528299 10.1002/alz.70363 PMC12173844

[6] Mayer CM, Belsham DD. Central insulin signaling is attenuated by long-term insulin exposure via insulin receptor substrate-1 serine phosphorylation, proteasomal degradation, and lysosomal insulin receptor degradation. Endocrinology. 2010;151(1):75–84. 19887566 10.1210/en.2009-0838

[7] Jeong S, Lin L, Leone AP, Hsu YH. Type 2 diabetes and late-onset Alzheimer’s disease and related dementia: a longitudinal cohort study integrating polygenic risk score. J Alzheimers Dis. 2025;105(1):107–19. 40129417 10.1177/13872877251326107

[8] Antal B, Mcmahon LP, Sultan SF, Lithen A, Wexler DJ, Dickerson B, et al. Type 2 diabetes mellitus accelerates brain aging and cognitive decline: complementary findings from UK Biobank and meta-analyses. Elife. 2022;11:e73138. 35608247 10.7554/eLife.73138 PMC9132576

[9] Xue M, Xu W, Ou YN, Cao XP, Tan MS, Tan L, et al. Diabetes mellitus and risks of cognitive impairment and dementia: A systematic review and meta-analysis of 144 prospective studies. Ageing Res Rev. 2019;55:100944. 31430566 10.1016/j.arr.2019.100944

[10] Chatterjee S, Peters SA, Woodward M, Mejia Arango S, Batty GD, Beckett N, et al. Type 2 diabetes as a risk factor for dementia in women compared with men: a pooled analysis of 2.3 million people comprising more than 100,000 cases of dementia. Diabetes Care. 2016;39(2):300–7. 26681727 10.2337/dc15-1588 PMC4722942

[11] Hogg E, Athreya K, Basile C, Tan EE, Kaminski J, Tagliati M. High prevalence of undiagnosed insulin resistance in non-diabetic subjects with Parkinson’s disease. J Parkinsons Dis. 2018;8(2):259–65. 29614702 10.3233/JPD-181305

[12] Faizan M, Sarkar A, Singh MP. Type 2 diabetes mellitus augments Parkinson’s disease risk or the other way around: Facts, challenges and future possibilities. Ageing Res Rev. 2022;81:101727. 36038113 10.1016/j.arr.2022.101727

[13] De Pablo-Fernandez E, Goldacre R, Pakpoor J, Noyce AJ, Warner TT. Association between diabetes and subsequent Parkinson disease: a record-linkage cohort study. Neurology. 2018;91(2):e139–42. 29898968 10.1212/WNL.0000000000005771

[14] Stockmann O, Ye L, Greten S, Chemodanow D, Wegner F, Klietz M. Impact of diabetes mellitus type two on incidence and progression of Parkinson’s disease: a systematic review of longitudinal patient cohorts. J Neural Transm. 2025;132(5):627–35. 39847072 10.1007/s00702-025-02882-7 PMC12043777

[15] Craft S, Watson GS. Insulin and neurodegenerative disease: shared and specific mechanisms. Lancet Neurol. 2004;3(3):169–78. 14980532 10.1016/S1474-4422(04)00681-7

[16] Chou SY, Chan L, Chung CC, Chiu JY, Hsieh YC, Hong CT. Altered insulin receptor substrate 1 phosphorylation in blood neuron-derived extracellular vesicles from patients with Parkinson’s Disease. Front Cell Dev Biol. 2020;8:564641. 33344443 10.3389/fcell.2020.564641 PMC7744811

[17] Leclerc M, Bourassa P, Tremblay C, Caron V, Sugère C, Emond V, et al. Cerebrovascular insulin receptors are defective in Alzheimer’s disease. Brain. 2023;146(1):75–90. 36280236 10.1093/brain/awac309 PMC9897197

[18] Shaughness M, Acs D, Brabazon F, Hockenbury N, Byrnes KR. Role of insulin in neurotrauma and neurodegeneration: a review. Front Neurosci. 2020;14:547175. 33100956 10.3389/fnins.2020.547175 PMC7546823

[19] Ansari MA, Al-Jarallah A, Babiker FA. Impaired insulin signaling alters mediators of hippocampal synaptic dynamics/plasticity: a possible mechanism of hyperglycemia-induced cognitive impairment. Cells. 2023;12(13):1728. 37443762 10.3390/cells12131728 PMC10340300

[20] Kakoty V, Kc S, Kumari S, Yang CH, Dubey SK, Sahebkar A, et al. Brain insulin resistance linked Alzheimer’s and Parkinson’s disease pathology: an undying implication of epigenetic and autophagy modulation. Inflammopharmacology. 2023;31(2):699–716. 36952096 10.1007/s10787-023-01187-z

[21] Scherer T, Sakamoto K, Buettner C. Brain insulin signalling in metabolic homeostasis and disease. Nat Rev Endocrinol. 2021;17(8):468–83. 34108679 10.1038/s41574-021-00498-x

[22] Kellar D, Craft S. Brain insulin resistance in Alzheimer’s disease and related disorders: mechanisms and therapeutic approaches. Lancet Neurol. 2020;19(9):758–66. 32730766 10.1016/S1474-4422(20)30231-3 PMC9661919

[23] Sun M, Mi W. Microglial insulin resistance drives neurodegeneration. Trends Endocrinol Metab. 2025;36(8):696–8. 40610268 10.1016/j.tem.2025.06.006

[24] Ekblad LL, Johansson J, Helin S, Viitanen M, Laine H, Puukka P, et al. Midlife insulin resistance, APOE genotype, and late-life brain amyloid accumulation. Neurology. 2018;90(13):e1150–7. 29476033 10.1212/WNL.0000000000005214 PMC5880630

[25] Rhea EM, Leclerc M, Yassine HN, Capuano AW, Tong H, Petyuk VA, et al. State of the science on brain insulin resistance and cognitive decline due to Alzheimer’s disease. Aging Dis. 2024;15(4):1688–725. 37611907 10.14336/AD.2023.0814 PMC11272209

[26] Choi E, Bai XC. The activation mechanism of the insulin receptor: a structural perspective. Annu Rev Biochem. 2023;92:247–72. 37001136 10.1146/annurev-biochem-052521-033250 PMC10398885

[27] Cormerais Y, Lapp SC, Kalafut KC, Cissé MY, Shin J, Stefadu B, et al. AKT-mediated phosphorylation of TSC2 controls stimulus- and tissue-specific mTORC1 signaling and organ growth. Dev Cell. 2025;60(19):2544-2557.e7. 40480230 10.1016/j.devcel.2025.05.008 PMC12258181

[28] Sanphui P, Biswas SC. FoxO3a is activated and executes neuron death via Bim in response to β-amyloid. Cell Death Dis. 2013;4(5):e625. 23661003 10.1038/cddis.2013.148 PMC3674357

[29] Chen W, Cai W, Hoover B, Kahn CR. Insulin action in the brain: cell types, circuits, and diseases. Trends Neurosci. 2022;45(5):384–400. 35361499 10.1016/j.tins.2022.03.001 PMC9035105

[30] Grillo CA, Piroli GG, Hendry RM, Reagan LP. Insulin-stimulated translocation of GLUT4 to the plasma membrane in rat hippocampus is PI3-kinase dependent. Brain Res. 2009;1296:35–45. 19679110 10.1016/j.brainres.2009.08.005 PMC2997526

[31] Frazier HN, Ghoweri AO, Anderson KL, Lin RL, Popa GJ, Mendenhall MD, et al. Elevating insulin signaling using a constitutively active insulin receptor increases glucose metabolism and expression of GLUT3 in hippocampal neurons. Front Neurosci. 2020;14:668. 32733189 10.3389/fnins.2020.00668 PMC7358706

[32] Chen Y, Qiu C, Yu W, Shao X, Zhou M, Wang Y. The relationship between brain glucose metabolism and insulin resistance in subjects with normal cognition - a study based on 18F-FDG PET. Nucl Med Commun. 2022;43(3):275–83. 34816810 10.1097/MNM.0000000000001511

[33] Baker LD, Cross DJ, Minoshima S, Belongia D, Watson GS, Craft S. Insulin resistance and Alzheimer-like reductions in regional cerebral glucose metabolism for cognitively normal adults with prediabetes or early type 2 diabetes. Arch Neurol. 2011;68(1):51–7. 20837822 10.1001/archneurol.2010.225 PMC3023149

[34] Zhao F, Siu JJ, Huang W, Askwith C, Cao L. Insulin modulates excitatory synaptic transmission and synaptic plasticity in the mouse hippocampus. Neuroscience. 2019;411:237–54. 31146008 10.1016/j.neuroscience.2019.05.033 PMC6612444

[35] Tang S, Liao Y, Yang M, Yue R. Brain insulin resistance: a key pathological hub linking metabolic and neuropsychiatric comorbidities. Front Aging Neurosci. 2026;18:1716291. 41878314 10.3389/fnagi.2026.1716291 PMC13006599

[36] Ramasubbu K, Devi Rajeswari V. Impairment of insulin signaling pathway PI3K/Akt/mTOR and insulin resistance induced AGEs on diabetes mellitus and neurodegenerative diseases: a perspective review. Mol Cell Biochem. 2023;478(6):1307–24. 36308670 10.1007/s11010-022-04587-x

[37] Kciuk M, Kruczkowska W, Gałęziewska J, Wanke K, Kałuzińska-Kołat Ż, Aleksandrowicz M, et al. Alzheimer’s disease as type 3 diabetes: understanding the link and implications. Int J Mol Sci. 2024;25(22):11955. 39596023 10.3390/ijms252211955 PMC11593477

[38] Hermann C, Assmus B, Urbich C, Zeiher AM, Dimmeler S. Insulin-mediated stimulation of protein kinase Akt: a potent survival signaling cascade for endothelial cells. Arterioscler Thromb Vasc Biol. 2000;20(2):402–9. 10669636 10.1161/01.atv.20.2.402

[39] Akhtar A, Sah SP. Insulin signaling pathway and related molecules: role in neurodegeneration and Alzheimer’s disease. Neurochem Int. 2020;135:104707. 32092326 10.1016/j.neuint.2020.104707

[40] Zakharova IO, Sokolova TV, Bayunova LV, Zorina II, Rychkova MP, Shpakov AO, et al. The protective effect of insulin on rat cortical neurons in oxidative stress and its dependence on the modulation of Akt, GSK-3beta, ERK1/2, and AMPK activities. Int J Mol Sci. 2019;20(15):3702. 31362343 10.3390/ijms20153702 PMC6696072

[41] Akamba Ambamba BD, Ella FA, Ngassa Ngoumen DJ, Dibacto Kemadjou RE, Agwe NI, Mbappe FE, et al. Tannins-enriched fraction of TeMac™ protects against aluminum chloride induced Alzheimer’s disease-like pathology by modulating aberrant insulin resistance and alleviating oxidative stress in diabetic rats. J Ethnopharmacol. 2024;335:118653. 39094753 10.1016/j.jep.2024.118653

[42] Böröczky C, Paszternák A, Laufer R, Tarnóczi K, Sikur N, Bagaméry F, et al. Neuroinflammation based neurodegenerative &lt;changed&gt;in vitro&lt;/changed&gt; model of SH-SY5Y cells-differential effects on oxidative stress and insulin resistance relevant to Alzheimer’s pathology. Int J Mol Sci. 2025;26(14):6581. 40724831 10.3390/ijms26146581 PMC12294424

[43] Frazier HN, Anderson KL, Ghoweri AO, Lin RL, Hawkinson TR, Popa GJ, et al. Molecular elevation of insulin receptor signaling improves memory recall in aged Fischer 344 rats. Aging Cell. 2020;19(10):e13220. 32852134 10.1111/acel.13220 PMC7576226

[44] Schiöth HB, Craft S, Brooks SJ, Frey WH 2nd, Benedict C. Brain insulin signaling and Alzheimer’s disease: current evidence and future directions. Mol Neurobiol. 2012;46(1):4–10. 22205300 10.1007/s12035-011-8229-6 PMC3443484

[45] Cui Y, Tang TY, Lu CQ, Ju S. Insulin resistance and cognitive impairment: evidence from neuroimaging. J Magn Reson Imaging. 2022;56(6):1621–49. 35852470 10.1002/jmri.28358

[46] Ni A, Ernst C. Evidence that substantia nigra pars compacta dopaminergic neurons are selectively vulnerable to oxidative stress because they are highly metabolically active. Front Cell Neurosci. 2022;16:826193. 35308118 10.3389/fncel.2022.826193 PMC8931026

[47] Unger J, Mcneill TH, Moxley RT 3rd, White M, Moss A, Livingston JN. Distribution of insulin receptor-like immunoreactivity in the rat forebrain. Neuroscience. 1989;31(1):143–57. 2771055 10.1016/0306-4522(89)90036-5

[48] Athauda D, Foltynie T. Insulin resistance and Parkinson’s disease: a new target for disease modification? Prog Neurobiol. 2016;145–146:98–120. 27713036 10.1016/j.pneurobio.2016.10.001

[49] Rivera EJ, Goldin A, Fulmer N, Tavares R, Wands JR, De La Monte SM. Insulin and insulin-like growth factor expression and function deteriorate with progression of Alzheimer’s disease: link to brain reductions in acetylcholine. J Alzheimers Dis. 2005;8(3):247–68. 16340083 10.3233/jad-2005-8304

[50] Ardanaz CG, De La Cruz A, Minhas PS, Hernández-Martín N, Pozo M, Valdecantos MP, et al. Astrocytic GLUT1 reduction paradoxically improves central and peripheral glucose homeostasis. Sci Adv. 2024;10(42):eadp1115. 39423276 10.1126/sciadv.adp1115 PMC11488540

[51] García-Cáceres C, Quarta C, Varela L, Gao Y, Gruber T, Legutko B, et al. Astrocytic insulin signaling couples brain glucose uptake with nutrient availability. Cell. 2016;166(4):867–80. 27518562 10.1016/j.cell.2016.07.028 PMC8961449

[52] Bonvento G, Bolaños JP. Astrocyte-neuron metabolic cooperation shapes brain activity. Cell Metab. 2021;33(8):1546–64. 34348099 10.1016/j.cmet.2021.07.006

[53] Bentivegna M, Pomilio C, Bellotto M, Pérez NG, Rossi SP, Gregosa A, et al. Amyloid beta regulates astrocytic glucose metabolism and insulin signaling in experimental models of Alzheimer’s Disease. Aging Dis. 2025. 10.14336/AD.2025.0484 40768640

[54] Robinson MB, Jackson JG. Astroglial glutamate transporters coordinate excitatory signaling and brain energetics. Neurochem Int. 2016;98:56–71. 27013346 10.1016/j.neuint.2016.03.014 PMC4969184

[55] Hascup ER, Broderick SO, Russell MK, Fang Y, Bartke A, Boger HA, et al. Diet-induced insulin resistance elevates hippocampal glutamate as well as VGLUT1 and GFAP expression in AβPP/PS1 mice. J Neurochem. 2019;148(2):219–37. 30472734 10.1111/jnc.14634 PMC6438176

[56] Das S, Mccloskey K, Nepal B, Kortagere S. EAAT2 activation regulates glutamate excitotoxicity and reduces impulsivity in a rodent model of Parkinson’s Disease. Mol Neurobiol. 2025;62(5):5787–803. 39630405 10.1007/s12035-024-04644-0 PMC11953204

[57] Tarantini S, Tran CHT, Gordon GR, Ungvari Z, Csiszar A. Impaired neurovascular coupling in aging and Alzheimer’s disease: contribution of astrocyte dysfunction and endothelial impairment to cognitive decline. Exp Gerontol. 2017;94:52–8. 27845201 10.1016/j.exger.2016.11.004 PMC5429210

[58] Tarantini S, Nyúl-Tóth Á, Yabluchanskiy A, Csipo T, Mukli P, Balasubramanian P, et al. Endothelial deficiency of insulin-like growth factor-1 receptor (IGF1R) impairs neurovascular coupling responses in mice, mimicking aspects of the brain aging phenotype. Geroscience. 2021;43(5):2387–94. 34383203 10.1007/s11357-021-00405-2 PMC8599783

[59] Fernandez AM, Martinez-Rachadell L, Navarrete M, Pose-Utrilla J, Davila JC, Pignatelli J, et al. Insulin regulates neurovascular coupling through astrocytes. Proc Natl Acad Sci U S A. 2022;119(29):e2204527119. 35858325 10.1073/pnas.2204527119 PMC9304019

[60] Canna A, Esposito F, Tedeschi G, Trojsi F, Passaniti C, Di Meo I, et al. Neurovascular coupling in patients with type 2 diabetes mellitus. Front Aging Neurosci. 2022;14:976340. 36118711 10.3389/fnagi.2022.976340 PMC9476313

[61] De Felice FG, Gonçalves RA, Ferreira ST. Impaired insulin signalling and allostatic load in Alzheimer disease. Nat Rev Neurosci. 2022;23(4):215–30. 35228741 10.1038/s41583-022-00558-9

[62] Raut S, Bhalerao A, Powers M, Gonzalez M, Mancuso S, Cucullo L. Hypometabolism, Alzheimer’s disease, and possible therapeutic targets: an overview. Cells. 2023;12(16):2019. 37626828 10.3390/cells12162019 PMC10453773

[63] Doust YV, Sumargo N, Ziebell JM, Premilovac D. Insulin resistance in the brain: evidence supporting a role for inflammation, reactive microglia, and the impact of biological sex. Neuroendocrinology. 2022;112(11):1027–38. 35279657 10.1159/000524059

[64] Darwish R, Alcibahy Y, Bucheeri S, Albishtawi A, Tama M, Shetty J, et al. The role of hypothalamic microglia in the onset of insulin resistance and type 2 diabetes: a neuro-immune perspective. Int J Mol Sci. 2024;25(23):13169. 39684879 10.3390/ijms252313169 PMC11642714

[65] Barone E, Butterfield DA. Insulin signaling in microglia: a metabolic switch controlling neuroinflammation and amyloid pathology in Alzheimer’s disease. Cell Metab. 2025;37(8):1630–2. 40769128 10.1016/j.cmet.2025.06.005

[66] Yang S, Qin C, Chen M, Chu YH, Tang Y, Zhou LQ, et al. TREM2-IGF1 mediated glucometabolic enhancement underlies microglial neuroprotective properties during ischemic stroke. Adv Sci (Weinh). 2024;11(10):e2305614. 38151703 10.1002/advs.202305614 PMC10933614

[67] Chen W, Liu X, Muñoz VR, Kahn CR. Loss of insulin signaling in microglia impairs cellular uptake of Aβ and neuroinflammatory response exacerbating AD-like neuropathology. Proc Natl Acad Sci U S A. 2025;122(21):e2501527122. 40388612 10.1073/pnas.2501527122 PMC12130885

[68] Fang M, Zhou Y, He K, Lu Y, Tao F, Huang H. Glucose metabolic reprogramming in microglia: implications for neurodegenerative diseases and targeted therapy. Mol Neurobiol. 2025;62(7):8204–21. 39987285 10.1007/s12035-025-04775-y

[69] Labandeira-Garcia JL, Costa-Besada MA, Labandeira CM, Villar-Cheda B, Rodríguez-Perez AI. Insulin-like growth factor-1 and neuroinflammation. Front Aging Neurosci. 2017;9:365. 29163145 10.3389/fnagi.2017.00365 PMC5675852

[70] Bassil F, Canron MH, Vital A, Bezard E, Li Y, Greig NH, et al. Insulin resistance and exendin-4 treatment for multiple system atrophy. Brain. 2017;140(5):1420–36. 28334990 10.1093/brain/awx044 PMC6248513

[71] De La Monte SM, Grammas P. Insulin resistance and oligodendrocyte/microvascular endothelial cell dysfunction as mediators of white matter degeneration in Alzheimer’s Disease. In: Wisniewski T, editor. Alzheimer’s disease. Brisbane (AU): The Authors; 2019. 31895517

[72] O’Grady JP, Dean DC 3rd, Yang KL, Canda CM, Hoscheidt SM, Starks EJ, et al. Elevated insulin and insulin resistance are associated with altered myelin in cognitively unimpaired middle-aged adults. Obesity (Silver Spring). 2019;27(9):1464–71. 31314172 10.1002/oby.22558 PMC6707894

[73] Liu CC, Hu J, Zhao N, Wang J, Wang N, Cirrito JR, et al. Astrocytic LRP1 mediates brain Aβ clearance and impacts amyloid deposition. J Neurosci. 2017;37(15):4023–31. 28275161 10.1523/JNEUROSCI.3442-16.2017 PMC5391682

[74] Storck SE, Meister S, Nahrath J, Meißner JN, Schubert N, Di Spiezio A, et al. Endothelial LRP1 transports amyloid-β(1-42) across the blood-brain barrier. J Clin Invest. 2016;126(1):123–36. 26619118 10.1172/JCI81108 PMC4701557

[75] Baltira C, Aronica E, Elmquist WF, Langer O, Löscher W, Sarkaria JN, et al. The impact of ATP-binding cassette transporters in the diseased brain: context matters. Cell Rep Med. 2024;5(6):101609. 38897176 10.1016/j.xcrm.2024.101609 PMC11228798

[76] Storck SE, Hartz AMS, Bernard J, Wolf A, Kachlmeier A, Mahringer A, et al. The concerted amyloid-beta clearance of LRP1 and ABCB1/P-gp across the blood-brain barrier is linked by PICALM. Brain Behav Immun. 2018;73:21–33. 30041013 10.1016/j.bbi.2018.07.017 PMC7748946

[77] Li W, Chen C, Xu B, Chen J, Yang M, Gao L, et al. The LDL Receptor-related protein 1: mechanisms and roles in promoting Aβ efflux transporter in Alzheimer’s disease. Biochem Pharmacol. 2025;231:116643. 39577706 10.1016/j.bcp.2024.116643

[78] Wang W, Bodles-Brakhop AM, Barger SW. A role for P-glycoprotein in clearance of Alzheimer amyloid β-peptide from the brain. Curr Alzheimer Res. 2016;13(6):615–20. 26971931 10.2174/1567205013666160314151012 PMC5102249

[79] Hong H, Liu LP, Liao JM, Wang TS, Ye FY, Wu J, et al. Downregulation of LRP1 [correction of LPR1] at the blood-brain barrier in streptozotocin-induced diabetic mice. Neuropharmacology. 2009;56(6–7):1054–9. 19285094 10.1016/j.neuropharm.2009.03.001

[80] Liu H, Liu X, Jia L, Liu Y, Yang H, Wang G, et al. Insulin therapy restores impaired function and expression of P-glycoprotein in blood-brain barrier of experimental diabetes. Biochem Pharmacol. 2008;75(8):1649–58. 18299117 10.1016/j.bcp.2008.01.004

[81] Cerasuolo M, Auriemma MC, Di Meo I, Lenti C, Papa M, Paolisso G, et al. Understanding the insulin-degrading enzyme: a new look at Alzheimer’s disease and Aβ plaque management. Int J Mol Sci. 2025;26(14):6693. 40724942 10.3390/ijms26146693 PMC12294565

[82] Portelius E, Mattsson N, Pannee J, Zetterberg H, Gisslén M, Vanderstichele H, et al. Ex vivo (18)O-labeling mass spectrometry identifies a peripheral amyloid β clearance pathway. Mol Neurodegener. 2017;12(1):18. 28219449 10.1186/s13024-017-0152-5 PMC5317049

[83] Yamamoto N, Ishikuro R, Tanida M, Suzuki K, Ikeda-Matsuo Y, Sobue K. Insulin-signaling pathway regulates the degradation of amyloid β-protein via astrocytes. Neuroscience. 2018;385:227–36. 29932983 10.1016/j.neuroscience.2018.06.018

[84] Kurochkin IV, Guarnera E, Berezovsky IN. Insulin-degrading enzyme in the fight against Alzheimer’s disease. Trends Pharmacol Sci. 2018;39(1):49–58. 29132916 10.1016/j.tips.2017.10.008

[85] Ly PT, Wu Y, Zou H, Wang R, Zhou W, Kinoshita A, et al. Inhibition of GSK3β-mediated BACE1 expression reduces Alzheimer-associated phenotypes. J Clin Invest. 2013;123(1):224–35. 23202730 10.1172/JCI64516 PMC3533290

[86] Yoon JH, Lee N, Youn K, Jo MR, Kim HR, Lee DS, et al. Dieckol ameliorates Aβ production via PI3K/Akt/GSK-3β regulated APP processing in SweAPP N2a cell. Mar Drugs. 2021;19(3):152. 33804171 10.3390/md19030152 PMC8001366

[87] Domoto T, Uehara M, Bolidong D, Minamoto T. Glycogen synthase kinase 3β in cancer biology and treatment. Cells. 2020;9(6):1388. 32503133 10.3390/cells9061388 PMC7349761

[88] Maixner DW, Weng HR. The role of glycogen synthase kinase 3 beta in neuroinflammation and pain. J Pharm Pharmacol (Los Angel). 2013;1(1):001. 25309941 10.13188/2327-204X.1000001 PMC4193379

[89] Huang J, Xu Z, Yu C, Liu L, Ji L, Qiu P, et al. The volatile oil of *Acorus tatarinowii* Schott ameliorates Alzheimer’s disease through improving insulin resistance via activating the PI3K/AKT pathway. Phytomedicine. 2024;135:156168. 39486109 10.1016/j.phymed.2024.156168

[90] Chatterjee S, Ambegaokar SS, Jackson GR, Mudher A. Insulin-mediated changes in tau hyperphosphorylation and autophagy in a *Drosophila* model of tauopathy and neuroblastoma cells. Front Neurosci. 2019;13:801. 31427921 10.3389/fnins.2019.00801 PMC6688711

[91] Leroy K, Yilmaz Z, Brion JP. Increased level of active GSK-3beta in Alzheimer’s disease and accumulation in argyrophilic grains and in neurones at different stages of neurofibrillary degeneration. Neuropathol Appl Neurobiol. 2007;33(1):43–55. 17239007 10.1111/j.1365-2990.2006.00795.x

[92] Hernandez F, Lucas JJ, Avila J. GSK3 and tau: two convergence points in Alzheimer’s disease. J Alzheimers Dis. 2013;33(Suppl 1):S141–4. 22710914 10.3233/JAD-2012-129025

[93] Talbot K, Wang HY, Kazi H, Han LY, Bakshi KP, Stucky A, et al. Demonstrated brain insulin resistance in Alzheimer’s disease patients is associated with IGF-1 resistance, IRS–1 dysregulation, and cognitive decline. J Clin Invest. 2012;122:1316–38. 22476197 10.1172/JCI59903 PMC3314463

[94] Oueslati A, Schneider BL, Aebischer P, Lashuel HA. Polo-like kinase 2 regulates selective autophagic α-synuclein clearance and suppresses its toxicity in vivo. Proc Natl Acad Sci U S A. 2013;110(41):E3945–54. 23983262 10.1073/pnas.1309991110 PMC3799334

[95] Hong CT, Chen KY, Wang W, Chiu JY, Wu D, Chao TY, et al. Insulin resistance promotes Parkinson’s disease through aberrant expression of α-synuclein, mitochondrial dysfunction, and deregulation of the polo-like kinase 2 signaling. Cells. 2020;9(3):740. 32192190 10.3390/cells9030740 PMC7140619

[96] Inglis KJ, Chereau D, Brigham EF, Chiou SS, Schöbel S, Frigon NL, et al. Polo-like kinase 2 (PLK2) phosphorylates alpha-synuclein at serine 129 in central nervous system. J Biol Chem. 2009;284(5):2598–602. 19004816 10.1074/jbc.C800206200 PMC2631975

[97] Chang YW, Hung LC, Chen YC, Wang WH, Lin CY, Tzeng HH, et al. Insulin reduces inflammation by regulating the activation of the NLRP3 inflammasome. Front Immunol. 2020;11:587229. 33679687 10.3389/fimmu.2020.587229 PMC7933514

[98] Alrouji M, Al-Kuraishy HM, Al-Gareeb AI, Alexiou A, Papadakis M, Jabir MS, et al. NF-κB/NLRP3 inflammasome axis and risk of Parkinson’s disease in Type 2 diabetes mellitus: a narrative review and new perspective. J Cell Mol Med. 2023;27(13):1775–89. 37210624 10.1111/jcmm.17784 PMC10315781

[99] De Pablo-Fernández E, Breen DP, Bouloux PM, Barker RA, Foltynie T, Warner TT. Neuroendocrine abnormalities in Parkinson’s disease. J Neurol Neurosurg Psychiatry. 2017;88(2):176–85. 27799297 10.1136/jnnp-2016-314601

[100] Chen L, Wang C, Qin L, Zhang H. Parkinson’s disease and glucose metabolism impairment. Transl Neurodegener. 2025;14(1):10. 39962629 10.1186/s40035-025-00467-8 PMC11831814

[101] Akter R, Cao P, Noor H, Ridgway Z, Tu LH, Wang H, et al. Islet amyloid polypeptide: structure, function, and pathophysiology. J Diabetes Res. 2016;2016:2798269. 26649319 10.1155/2016/2798269 PMC4662979

[102] Marmentini C, Branco RCS, Boschero AC, Kurauti MA. Islet amyloid toxicity: from genesis to counteracting mechanisms. J Cell Physiol. 2022;237(2):1119–42. 34636428 10.1002/jcp.30600

[103] Zhang G, Meng L, Wang Z, Peng Q, Chen G, Xiong J, et al. Islet amyloid polypeptide cross-seeds tau and drives the neurofibrillary pathology in Alzheimer’s disease. Mol Neurodegener. 2022;17(1):12. 35093145 10.1186/s13024-022-00518-y PMC8800231

[104] Libard S, Alafuzoff I. Is islet amyloid polypeptide indeed expressed in the human brain. Neuropathol Appl Neurobiol. 2023;49(4):e12917. 37317631 10.1111/nan.12917

[105] Dharmaraj GL, Arigo FD, Young KA, Martins R, Mancera RL, Bharadwaj P. Novel amylin analogues reduce amyloid-β cross-seeding aggregation and neurotoxicity. J Alzheimers Dis. 2022;87(1):373–90. 35275530 10.3233/JAD-215339

[106] Krotee P, Griner SL, Sawaya MR, Cascio D, Rodriguez JA, Shi D, et al. Common fibrillar spines of amyloid-β and human islet amyloid polypeptide revealed by microelectron diffraction and structure-based inhibitors. J Biol Chem. 2018;293(8):2888–902. 29282295 10.1074/jbc.M117.806109 PMC5827424

[107] Arya S, Claud SL, Cantrell KL, Bowers MT. Catalytic prion-like cross-talk between a key Alzheimer’s disease tau-fragment R3 and the type 2 diabetes peptide IAPP. ACS Chem Neurosci. 2019;10(11):4757–65. 31642657 10.1021/acschemneuro.9b00516

[108] Santos N, Segura L, Lewis A, Pham T, Cheng K-O. Multiscale modeling of macromolecular interactions between tau-amylin oligomers and asymmetric lipid nanodomains that link Alzheimer’s and diabetic diseases. Molecules. 2024;29(3):740. 38338484 10.3390/molecules29030740 PMC10856442

[109] Mucibabic M, Steneberg P, Lidh E, Straseviciene J, Ziolkowska A, Dahl U, et al. α-Synuclein promotes IAPP fibril formation in vitro and β-cell amyloid formation in vivo in mice. Sci Rep. 2020;10(1):20438. 33235246 10.1038/s41598-020-77409-z PMC7686322

[110] Horvath I, Wittung-Stafshede P. Cross-talk between amyloidogenic proteins in type-2 diabetes and Parkinson’s disease. Proc Natl Acad Sci U S A. 2016;113(44):12473–7. 27791129 10.1073/pnas.1610371113 PMC5098634

[111] Zhou Y, Lai M, Shu B, Wang B, Wang D, Liu H, et al. Unraveling α-synuclein and amylin co-aggregation: pathological insights and biomarker development for Parkinson’s disease. Theranostics. 2025;15(15):7409–24. 40756347 10.7150/thno.112396 PMC12315819

[112] Meng L, Li Y, Liu C, Zhang G, Chen J, Xiong M, et al. Islet amyloid polypeptide triggers α-synuclein pathology in Parkinson’s disease. Prog Neurobiol. 2023;226:102462. 37150314 10.1016/j.pneurobio.2023.102462

[113] Martinez-Valbuena I, Valenti-Azcarate R, Amat-Villegas I, Riverol M, Marcilla I, De Andrea CE, et al. Amylin as a potential link between type 2 diabetes and alzheimer disease. Ann Neurol. 2019;86(4):539–51. 31376172 10.1002/ana.25570

[114] Martinez-Valbuena I, Amat-Villegas I, Valenti-Azcarate R, Carmona-Abellan MDM, Marcilla I, Tuñon MT, et al. Interaction of amyloidogenic proteins in pancreatic β cells from subjects with synucleinopathies. Acta Neuropathol. 2018;135(6):877–86. 29536165 10.1007/s00401-018-1832-0

[115] Ly H, Verma N, Sharma S, Kotiya D, Despa S, Abner EL, et al. The association of circulating amylin with β-amyloid in familial Alzheimer’s disease. Alzheimers Dement (N Y). 2021;7(1):e12130. 33521236 10.1002/trc2.12130 PMC7816817

[116] Chen HC, Cao JX, Cai YT, Du HL, Xi XX, Sun J, et al. Interaction of human IAPP and Aβ(1)-(42) aggravated the AD-related pathology and impaired the cognition in mice. Exp Neurol. 2020;334:113490. 33007295 10.1016/j.expneurol.2020.113490

[117] Caiyan L, Feng Y. The deposition and cross-seeding of islet amyloid polypeptide and phosphorylated α-synuclein in gut, myocardium, and brain of cynomolgus monkeys with spontaneous type 2 diabetes mellitus. In: Proc SPIE. 2025; 13296: 132960F.

[118] Sun Y, Guo C, Yuan L, Li W, Wang ZY, Yue F, et al. Cynomolgus monkeys with spontaneous type-2-diabetes-mellitus-like pathology develop alpha-synuclein alterations reminiscent of prodromal Parkinson’s disease and related diseases. Front Neurosci. 2020;14:63. 32116510 10.3389/fnins.2020.00063 PMC7019001

[119] Wijesekara N, Ahrens R, Sabale M, Wu L, Ha K, Verdile G, et al. Amyloid-β and islet amyloid pathologies link Alzheimer’s disease and type 2 diabetes in a transgenic model. FASEB J. 2017;31(12):5409–18. 28808140 10.1096/fj.201700431R

[120] Wijesekara N, Gonçalves RA, Ahrens R, Ha K, De Felice FG, Fraser PE. Combination of human tau and islet amyloid polypeptide exacerbates metabolic dysfunction in transgenic mice. J Pathol. 2021;254(3):244–53. 33797777 10.1002/path.5674

[121] Wong CYJ, Baldelli A, Tietz O, Leite E, Ong HX, Traini D. Intranasal delivery of insulin: an update on status quo and challenges for diabetes treatment. Int J Biol Macromol. 2025;332(Pt 2):148663. 41177483 10.1016/j.ijbiomac.2025.148663

[122] Chapman CD, Schiöth HB, Grillo CA, Benedict C. Intranasal insulin in Alzheimer’s disease: food for thought. Neuropharmacology. 2018;136(Pt B):196–201. 29180222 10.1016/j.neuropharm.2017.11.037 PMC10523803

[123] Lochhead JJ, Kellohen KL, Ronaldson PT, Davis TP. Distribution of insulin in trigeminal nerve and brain after intranasal administration. Sci Rep. 2019;9(1):2621. 30796294 10.1038/s41598-019-39191-5 PMC6385374

[124] Gabbouj S, Ryhänen S, Marttinen M, Wittrahm R, Takalo M, Kemppainen S, et al. Altered insulin signaling in Alzheimer’s disease brain—special emphasis on PI3K-Akt pathway. Front Neurosci. 2019;13:629. 31275108 10.3389/fnins.2019.00629 PMC6591470

[125] Kellar D, Register T, Lockhart SN, Aisen P, Raman R, Rissman RA, et al. Intranasal insulin modulates cerebrospinal fluid markers of neuroinflammation in mild cognitive impairment and Alzheimer’s disease: a randomized trial. Sci Rep. 2022;12(1):1346. 35079029 10.1038/s41598-022-05165-3 PMC8789895

[126] Woodfield A, Gonzales T, Helmerhorst E, Laws S, Newsholme P, Porter T, et al. Current insights on the use of insulin and the potential use of insulin mimetics in targeting insulin signalling in Alzheimer’s disease. Int J Mol Sci. 2022;23(24):15811. 36555450 10.3390/ijms232415811 PMC9779379

[127] Erichsen JM, Calva CB, Reagan LP, Fadel JR. Intranasal insulin and orexins to treat age-related cognitive decline. Physiol Behav. 2021;234:113370. 33621561 10.1016/j.physbeh.2021.113370 PMC8053680

[128] Duarte AI, Santos P, Oliveira CR, Santos MS, Rego AC. Insulin neuroprotection against oxidative stress is mediated by Akt and GSK-3beta signaling pathways and changes in protein expression. Biochim Biophys Acta. 2008;1783(6):994–1002. 18348871 10.1016/j.bbamcr.2008.02.016

[129] Rajasekar N, Nath C, Hanif K, Shukla R. Intranasal insulin administration ameliorates streptozotocin (ICV)-induced insulin receptor dysfunction, neuroinflammation, amyloidogenesis, and memory impairment in rats. Mol Neurobiol. 2017;54(8):6507–22. 27730514 10.1007/s12035-016-0169-8

[130] Mao YF, Guo Z, Zheng T, Jiang Y, Yan Y, Yin X, et al. Intranasal insulin alleviates cognitive deficits and amyloid pathology in young adult APPswe/PS1dE9 mice. Aging Cell. 2016;15(5):893–902. 27457264 10.1111/acel.12498 PMC5013027

[131] Guo Z, Chen Y, Mao YF, Zheng T, Jiang Y, Yan Y, et al. Long-term treatment with intranasal insulin ameliorates cognitive impairment, tau hyperphosphorylation, and microglial activation in a streptozotocin-induced Alzheimer’s rat model. Sci Rep. 2017;7:45971. 28382978 10.1038/srep45971 PMC5382700

[132] Chen Y, Zhao Y, Dai CL, Liang Z, Run X, Iqbal K, et al. Intranasal insulin restores insulin signaling, increases synaptic proteins, and reduces Aβ level and microglia activation in the brains of 3xTg-AD mice. Exp Neurol. 2014;261:610–9. 24918340 10.1016/j.expneurol.2014.06.004

[133] Mao YF, Chen L, Liu JY, Guo ZY, Lu PL, Chen YX. Intranasal insulin administration shows limited tau-targeted effects in early-stage APP/PS1 Alzheimer’s mice. Neurosci Lett. 2025;865:138337. 40759310 10.1016/j.neulet.2025.138337

[134] De Oliveira Andrade LJ, Matos G, Matos De Oliveira L. Intranasal insulin in Alzheimer disease (diabetes in situ?): a systematic review and meta-analysis. Dement Neuropsychol. 2025;19:20240191. 10.1590/1980-5764-DN-2024-0191 PMC11975293 40195962

[135] Craft S, Raman R, Chow TW, Rafii MS, Sun CK, Rissman RA, et al. Safety, efficacy, and feasibility of intranasal insulin for the treatment of mild cognitive impairment and Alzheimer disease dementia: a randomized clinical trial. JAMA Neurol. 2020;77(9):1099–109. 32568367 10.1001/jamaneurol.2020.1840 PMC7309571

[136] Schmid V, Kullmann S, Gfrörer W, Hund V, Hallschmid M, Lipp HP, et al. Safety of intranasal human insulin: a review. Diabetes Obes Metab. 2018;20(7):1563–77. 29508509 10.1111/dom.13279

[137] Claxton A, Baker LD, Wilkinson CW, Trittschuh EH, Chapman D, Watson GS, et al. Sex and ApoE genotype differences in treatment response to two doses of intranasal insulin in adults with mild cognitive impairment or Alzheimer’s disease. J Alzheimers Dis. 2013;35(4):789–97. 23507773 10.3233/JAD-122308 PMC4144993

[138] Picone P, Sabatino MA, Ditta LA, Amato A, San Biagio PL, Mulè F, et al. Nose-to-brain delivery of insulin enhanced by a nanogel carrier. J Control Release. 2018;270:23–36. 29196041 10.1016/j.jconrel.2017.11.040

[139] Picone P, Ditta LA, Sabatino MA, Militello V, San Biagio PL, Di Giacinto ML, et al. Ionizing radiation-engineered nanogels as insulin nanocarriers for the development of a new strategy for the treatment of Alzheimer’s disease. Biomaterials. 2016;80:179–94. 26708643 10.1016/j.biomaterials.2015.11.057

[140] Georgiou A, Zanos P, Onisiforou A. Metformin provides superior neuroprotective potential compared to semaglutide in preventing diabetes-associated Alzheimer’s disease via dual actions. Commun Med (Lond). 2026;6(1):196. 41760788 10.1038/s43856-026-01471-3 PMC13066627

[141] Cui W, Lv C, Geng P, Fu M, Zhou W, Xiong M, et al. Novel targets and therapies of metformin in dementia: old drug, new insights. Front Pharmacol. 2024;15:1415740. 38881878 10.3389/fphar.2024.1415740 PMC11176471

[142] Katila N, Bhurtel S, Park PH, Hong JT, Choi DY. Activation of AMPK/aPKCζ/CREB pathway by metformin is associated with upregulation of GDNF and dopamine. Biochem Pharmacol. 2020;180:114193. 32800853 10.1016/j.bcp.2020.114193

[143] Mary A, Barale S, Eysert F, Valverde A, Lacas-Gervais S, Bauer C, et al. Hampered AMPK-ULK1 cascade in Alzheimer’s disease (AD) instigates mitochondria dysfunctions and AD-related alterations which are alleviated by metformin. Alzheimers Res Ther. 2025;17(1):127. 40457477 10.1186/s13195-025-01772-0 PMC12128297

[144] Alves SS, Rossi L, De Oliveira JAC, Servilha-Menezes G, Grigorio-De-Sant’Ana M, Mazzei RF, et al. Metformin improves spatial memory and reduces seizure severity in a rat model of epilepsy and Alzheimer’s disease comorbidity via PI3K/Akt signaling pathway. Mol Neurobiol. 2025;62(8):9545–72. 40126600 10.1007/s12035-025-04844-2

[145] Ou Z, Kong X, Sun X, He X, Zhang L, Gong Z, et al. Metformin treatment prevents amyloid plaque deposition and memory impairment in APP/PS1 mice. Brain Behav Immun. 2018;69:351–63. 29253574 10.1016/j.bbi.2017.12.009

[146] Katila N, Bhurtel S, Shadfar S, Srivastav S, Neupane S, Ojha U, et al. Metformin lowers α-synuclein phosphorylation and upregulates neurotrophic factor in the MPTP mouse model of Parkinson’s disease. Neuropharmacology. 2017;125:396–407. 28807678 10.1016/j.neuropharm.2017.08.015

[147] Lu M, Su C, Qiao C, Bian Y, Ding J, Hu G. Metformin prevents dopaminergic neuron death in MPTP/P-induced mouse model of Parkinson’s disease via autophagy and mitochondrial ROS clearance. Int J Neuropsychopharmacol. 2016;19(9):pyw047. 27207919 10.1093/ijnp/pyw047 PMC5043649

[148] Sun M, Wang X, Lu Z, Yang Y, Lv S, Miao M, et al. Metformin use and risk of delirium in older adults with type 2 diabetes. Diabetes Care. 2025;48(7):1172–9. 39361020 10.2337/dc24-1414

[149] Zheng B, Su B, Ahmadi-Abhari S, Kapogiannis D, Tzoulaki I, Riboli E, et al. Dementia risk in patients with type 2 diabetes: comparing metformin with no pharmacological treatment. Alzheimers Dement. 2023;19(12):5681–9. 37395154 10.1002/alz.13349 PMC12180072

[150] Nair KS, Pataky M, Ruegsegger G, Jo HJ, Klaus K, Sevits K, et al. Improvement in insulin sensitivity prevents decline in glucose uptake, functional connectivity, and volume in the insulin resistant human brain. Res Sq. 2025.

[151] Yang AJT, Frendo-Cumbo S, Macpherson REK. Resveratrol and metformin recover prefrontal cortex AMPK activation in diet-induced obese mice but reduce BDNF and synaptophysin protein content. J Alzheimers Dis. 2019;71(3):945–56. 31450493 10.3233/JAD-190123

[152] Howell JJ, Hellberg K, Turner M, Talbott G, Kolar MJ, Ross DS, et al. Metformin inhibits hepatic mTORC1 signaling via dose-dependent mechanisms involving AMPK and the TSC complex. Cell Metab. 2017;25(2):463–71. 28089566 10.1016/j.cmet.2016.12.009 PMC5299044

[153] Nicol NI, Ma T. Activation of the AMPK signaling and brain function: a friend or foe? J Neurochem. 2025;169(11):e70283. 41194471 10.1111/jnc.70283 PMC13421047

[154] Domise M, Sauvé F, Didier S, Caillerez R, Bégard S, Carrier S, et al. Neuronal AMP-activated protein kinase hyper-activation induces synaptic loss by an autophagy-mediated process. Cell Death Dis. 2019;10(3):221. 30833547 10.1038/s41419-019-1464-x PMC6399353

[155] Weisová P, Dávila D, Tuffy LP, Ward MW, Concannon CG, Prehn JH. Role of 5’-adenosine monophosphate-activated protein kinase in cell survival and death responses in neurons. Antioxid Redox Signal. 2011;14(10):1863–76. 20712420 10.1089/ars.2010.3544

[156] Curry DW, Stutz B, Andrews ZB, Elsworth JD. Targeting AMPK signaling as a neuroprotective strategy in Parkinson’s disease. J Parkinsons Dis. 2018;8(2):161–81. 29614701 10.3233/JPD-171296 PMC6004921

[157] Kruczkowska W, Gałęziewska J, Buczek P, Płuciennik E, Kciuk M, Śliwińska A. Overview of metformin and neurodegeneration: a comprehensive review. Pharmaceuticals (Basel). 2025;18(4):486. 40283923 10.3390/ph18040486 PMC12030719

[158] Bernardo A, Minghetti L. PPAR-gamma agonists as regulators of microglial activation and brain inflammation. Curr Pharm Des. 2006;12(1):93–109. 16454728 10.2174/138161206780574579

[159] Khan MA, Alam Q, Haque A, Ashafaq M, Khan MJ, Ashraf GM, et al. Current progress on peroxisome proliferator-activated receptor gamma agonist as an emerging therapeutic approach for the treatment of Alzheimer’s disease: an update. Curr Neuropharmacol. 2019;17(3):232–46. 30152284 10.2174/1570159X16666180828100002 PMC6425074

[160] Zu J, Li C, Cui M, Liu X, Pan Z, Li X, et al. Pioglitazone attenuates complement-mediated microglial synaptic engulfment in an Alzheimer’s disease model. Brain. 2026;149(2):668–79. 41396874 10.1093/brain/awaf462

[161] Quan Q, Qian Y, Li X, Li M. Pioglitazone reduces β amyloid levels via inhibition of PPARγ phosphorylation in a neuronal model of Alzheimer’s disease. Front Aging Neurosci. 2019;11:178. 31379559 10.3389/fnagi.2019.00178 PMC6650543

[162] Santiago-Balmaseda A, Villegas-Rojas MM, Pérez-Segura I, Espino-Cambrón M, Martinez-Fong D, Soto-Rojas LO. Schedule-dependent neuroprotection by pioglitazone in a novel model of α-synucleinopathy in rats: integrated behavioural and histological outcomes. Br J Pharmacol. 2026;183(15):4227-4245. 42033189 10.1111/bph.70431

[163] Alhowail A, Alsikhan R, Alsaud M, Aldubayan M, Rabbani SI. Protective effects of pioglitazone on cognitive impairment and the underlying mechanisms: a review of literature. Drug Des Devel Ther. 2022;16:2919–31. 36068789 10.2147/DDDT.S367229 PMC9441149

[164] Tseng CH. Pioglitazone reduces dementia risk in patients with type 2 diabetes mellitus: a retrospective cohort analysis. J Clin Med. 2018;7(10):306. 30262775 10.3390/jcm7100306 PMC6209987

[165] Burns DK, Alexander RC, Welsh-Bohmer KA, Culp M, Chiang C, O’Neil J, et al. Safety and efficacy of pioglitazone for the delay of cognitive impairment in people at risk of Alzheimer’s disease (TOMMORROW): a prognostic biomarker study and a phase 3, randomised, double-blind, placebo-controlled trial. Lancet Neurol. 2021;20(7):537–47. 34146512 10.1016/S1474-4422(21)00043-0

[166] Nelson ML, Pfeifer JA, Hickey JP, Collins AE, Kalisch BE. Exploring rosiglitazone’s potential to treat Alzheimer’s disease through the modulation of brain-derived neurotrophic factor. Biology (Basel). 2023;12(7):1042. 37508471 10.3390/biology12071042 PMC10376118

[167] Deo AK, Theil FP, Nicolas JM. Confounding parameters in preclinical assessment of blood-brain barrier permeation: an overview with emphasis on species differences and effect of disease states. Mol Pharm. 2013;10(5):1581–95. 23256608 10.1021/mp300570z

[168] Wallach JD, Wang K, Zhang AD, Cheng D, Grossetta Nardini HK, Lin H, et al. Updating insights into rosiglitazone and cardiovascular risk through shared data: individual patient and summary level meta-analyses. BMJ. 2020;368:l7078. 32024657 10.1136/bmj.l7078 PMC7190063

[169] Tuccori M, Filion KB, Yin H, Yu OH, Platt RW, Azoulay L. Pioglitazone use and risk of bladder cancer: population based cohort study. BMJ. 2016;352:i1541. 27029385 10.1136/bmj.i1541 PMC4816602

[170] Govindan J, Evans M. Pioglitazone in clinical practice: where are we now? Diabetes Ther. 2012;3(1):1–8. 22373598 10.1007/s13300-012-0001-z PMC3508118

[171] Hölscher C. Novel dual GLP-1/GIP receptor agonists show neuroprotective effects in Alzheimer’s and Parkinson’s disease models. Neuropharmacology. 2018;136(Pt B):251–9. 29402504 10.1016/j.neuropharm.2018.01.040

[172] Kopp KO, Glotfelty EJ, Li Y, Greig NH. Glucagon-like peptide-1 (GLP-1) receptor agonists and neuroinflammation: implications for neurodegenerative disease treatment. Pharmacol Res. 2022;186:106550. 36372278 10.1016/j.phrs.2022.106550 PMC9712272

[173] Katsurada K, Yada T. Neural effects of gut- and brain-derived glucagon-like peptide-1 and its receptor agonist. J Diabetes Investig. 2016;7(Suppl 1):64–9. 27186358 10.1111/jdi.12464 PMC4854507

[174] Kang X, Wang D, Zhang L, Huang T, Liu S, Feng X, et al. Exendin-4 ameliorates tau hyperphosphorylation and cognitive impairment in type 2 diabetes through acting on Wnt/β-catenin/NeuroD1 pathway. Mol Med. 2023;29(1):118. 37667187 10.1186/s10020-023-00718-2 PMC10478475

[175] Tian S, Tan S, Jia W, Zhao J, Sun X. Activation of Wnt/β-catenin signaling restores insulin sensitivity in insulin resistant neurons through transcriptional regulation of IRS-1. J Neurochem. 2021;157(3):467–78. 33336396 10.1111/jnc.15277

[176] Jantrapirom S, Nimlamool W, Chattipakorn N, Chattipakorn S, Temviriyanukul P, Inthachat W, et al. Liraglutide suppresses tau hyperphosphorylation, amyloid beta accumulation through regulating neuronal insulin signaling and BACE-1 activity. Int J Mol Sci. 2020;21(5):1725. 32138327 10.3390/ijms21051725 PMC7084306

[177] Ma DL, Chen FQ, Xu WJ, Yue WZ, Yuan G, Yang Y. Early intervention with glucagon-like peptide 1 analog liraglutide prevents tau hyperphosphorylation in diabetic db/db mice. J Neurochem. 2015;135(2):301–8. 26183127 10.1111/jnc.13248

[178] Wang ZJ, Li XR, Chai SF, Li WR, Li S, Hou M, et al. Semaglutide ameliorates cognition and glucose metabolism dysfunction in the 3xTg mouse model of Alzheimer’s disease via the GLP-1R/SIRT1/GLUT4 pathway. Neuropharmacology. 2023;240:109716. 37730113 10.1016/j.neuropharm.2023.109716

[179] Li L, Cao D, Lu H, Lewis T, Mans R. O3-05-03: Glucagon-like peptide-1 mimetic peptide Exendin 4 preserves memory function and attenuates neuropathology in APP/PS1 double transgenic mice. Alzheimers Dement. 2010;6(4S_Part_5):S135.

[180] Bu LL, Liu YQ, Shen Y, Fan Y, Yu WB, Jiang DL, et al. Neuroprotection of exendin-4 by enhanced autophagy in a parkinsonian rat model of α-synucleinopathy. Neurotherapeutics. 2021;18(2):962–78. 33723752 10.1007/s13311-021-01018-5 PMC8423983

[181] Reiner J, Thiery J, Held J, Berlin P, Skarbaliene J, Vollmar B, et al. The dual GLP-1 and GLP-2 receptor agonist dapiglutide promotes barrier function in murine short bowel. Ann N Y Acad Sci. 2022;1514(1):132–41. 35580981 10.1111/nyas.14791

[182] Bang-Berthelsen CH, Holm TL, Pyke C, Simonsen L, Søkilde R, Pociot F, et al. GLP-1 induces barrier protective expression in Brunner’s glands and regulates colonic inflammation. Inflamm Bowel Dis. 2016;22(9):2078–97. 27542128 10.1097/MIB.0000000000000847

[183] Su Y, Liu N, Zhang Z, Li H, Ma J, Yuan Y, et al. Cholecystokinin and glucagon-like peptide-1 analogues regulate intestinal tight junction, inflammation, dopaminergic neurons and α-synuclein accumulation in the colon of two Parkinson’s disease mouse models. Eur J Pharmacol. 2022;926:175029. 35584709 10.1016/j.ejphar.2022.175029

[184] Athauda D, Maclagan K, Skene SS, Bajwa-Joseph M, Letchford D, Chowdhury K, et al. Exenatide once weekly versus placebo in Parkinson’s disease: a randomised, double-blind, placebo-controlled trial. Lancet. 2017;390(10103):1664–75. 28781108 10.1016/S0140-6736(17)31585-4 PMC5831666

[185] Engström A, Svanström H, Hviid A, Eliasson B, Gudbjörnsdottir S, Wintzell V, et al. Use of glucagon-like peptide-1 receptor agonists and risk of Parkinson’s Disease: Scandinavian cohort study. Diabetes Obes Metab. 2026;28(7):5767–5778. 41994910 10.1111/dom.70760 PMC13243990

[186] Schechter M, Fishkin A, Mosenzon O, Sehtman-Shachar DR, Cukierman-Yaffe T, Leibowitz G, et al. Neurodegeneration onset with glucagon-like peptide-1 receptor agonists in people with type 2 diabetes: a real-world multinational cohort study. Cardiovasc Diabetol. 2025;24(1):426. 41204243 10.1186/s12933-025-02962-8 PMC12595915

[187] Edison P, Femminella GD, Ritchie C, Nowell J, Holmes C, Walker Z, et al. Liraglutide in mild to moderate Alzheimer’s disease: a phase 2b clinical trial. Nat Med. 2026;32(1):353–61. 41326666 10.1038/s41591-025-04106-7 PMC12823385

[188] Wang W, Tanokashira D, Fukui Y, Maruyama M, Kuroiwa C, Saito T, et al. Serine phosphorylation of IRS1 correlates with Aβ-unrelated memory deficits and elevation in Aβ level prior to the onset of memory decline in AD. Nutrients. 2019;11(8):1942. 31426549 10.3390/nu11081942 PMC6723493

[189] Komleva YK, Potapenko IV, Lopatina OL, Gorina YV, Chernykh A, Khilazheva ED, et al. NLRP3 inflammasome blocking as a potential treatment of central insulin resistance in early-stage Alzheimer’s disease. Int J Mol Sci. 2021;22(21):11588. 34769018 10.3390/ijms222111588 PMC8583950

[190] Ghuman M, Boutajangout A, Atta A, Kaddioui RE, Kozikowski AP, Wisniewski T. Efficacy of an inhibitor of GSK-3β in an Alzheimer’s disease mouse model. Alzheimers Dement. 2024;20(Suppl 1):e093368.

[191] Manolopoulos KN, Klotz LO, Korsten P, Bornstein SR, Barthel A. Linking Alzheimer’s disease to insulin resistance: the FoxO response to oxidative stress. Mol Psychiatry. 2010;15(11):1046–52. 20966918 10.1038/mp.2010.17

[192] Kaur P, Khan H, Grewal AK, Dua K, Singh SK, Gupta G, et al. Exploring therapeutic strategies: the relationship between metabolic disorders and FOXO signalling in Alzheimer’s disease. CNS Neurol Disord Drug Targets. 2025;24(3):196–207. 39473249 10.2174/0118715273321002240919102841

[193] Sajan M, Hansen B, Ivey R 3rd, Sajan J, Ari C, Song S, et al. Brain insulin signaling is increased in insulin-resistant states and decreases in FOXOs and PGC-1α and increases in Aβ1-40/42 and phospho-tau may abet Alzheimer development. Diabetes. 2016;65(7):1892–903. 26895791 10.2337/db15-1428 PMC4915579

[194] Davidy T, Yore I, Cukierman-Yaffe T, Ravona-Springer R, Livny A, Lesman-Segev OH, et al. A feasibility study of the combination of intranasal insulin with oral semaglutide for cognition in older adults with metabolic syndrome at high dementia risk- study rationale and design. Mech Ageing Dev. 2024;218:111898. 38159613 10.1016/j.mad.2023.111898

[195] Zhang L, Zhang L, Li Y, Li L, Melchiorsen JU, Rosenkilde M, et al. The novel dual GLP-1/GIP receptor agonist DA-CH5 is superior to single GLP-1 receptor agonists in the MPTP model of Parkinson’s disease. J Parkinsons Dis. 2020;10(2):523–42. 31958096 10.3233/JPD-191768

[196] Feng P, Liu Z, Lv D, Hao W, Li D, Xue G, et al. The novel GLP-1 / GIP dual agonist DA3-CH is more effective than liraglutide in the MPTP mouse model of Parkinson’s disease. Eur J Pharmacol. 2025;1003:177972. 40683437 10.1016/j.ejphar.2025.177972

[197] Hölscher C. Incretin hormones GLP-1 and GIP normalize energy utilization and reduce inflammation in the brain in Alzheimer’s Disease and Parkinson’s Disease: from repurposed GLP-1 receptor agonists to novel dual GLP-1/GIP receptor agonists as potential disease-modifying therapies. CNS Drugs. 2025;39(12):1201–20. 40938528 10.1007/s40263-025-01226-z PMC12602575

[198] Yang J, Shi X, Wang Y, Ma M, Liu H, Wang J, et al. Multi-target neuroprotection of thiazolidinediones on Alzheimer’s Disease via neuroinflammation and ferroptosis. J Alzheimers Dis. 2023;96(3):927–45. 37927258 10.3233/JAD-230593 PMC10741341

[199] Reczek CR, Chakrabarty RP, D’Alessandro KB, Sebo ZL, Grant RA, Gao P, et al. Metformin targets mitochondrial complex I to lower blood glucose levels. Sci Adv. 2024;10(51):eads5466. 39693440 10.1126/sciadv.ads5466 PMC11654692

[200] Shang H, Wang Z, Sun Y, Zuo C, Wang M, Zheng K, et al. Metformin inhibits microglial activation-mediated cuproptosis by modulating the TLR4/Myd88/NF-κB signaling pathway in Parkinson’s Disease. Mol Neurobiol. 2025;63(1):95. 41261257 10.1007/s12035-025-05499-9 PMC12630286

[201] Zheng Z, Bian Y, Zhang Y, Ren G, Li G. Metformin activates AMPK/SIRT1/NF-κB pathway and induces mitochondrial dysfunction to drive caspase3/GSDME-mediated cancer cell pyroptosis. Cell Cycle. 2020;19(10):1089–104. 32286137 10.1080/15384101.2020.1743911 PMC7217368

[202] Qiu L, Jiang X, Wen L, Hu Q, Deng Y. Pioglitazone decreases the levels of inflammatory cytokines in SD rats with traumatic brain injury via up-regulating PPARγ. Xi Bao Yu Fen Zi Mian Yi Xue Za Zhi. 2016;32(2):182–4. 26927377

[203] Sadek MA, Kandil EA, El Sayed NS, Sayed HM, Rabie MA. Semaglutide, a novel glucagon-like peptide-1 agonist, amends experimental autoimmune encephalomyelitis-induced multiple sclerosis in mice: involvement of the PI3K/Akt/GSK-3β pathway. Int Immunopharmacol. 2023;115:109647. 36584570 10.1016/j.intimp.2022.109647

[204] Reger MA, Watson GS, Green PS, Wilkinson CW, Baker LD, Cholerton B, et al. Intranasal insulin improves cognition and modulates beta-amyloid in early AD. Neurology. 2008;70(6):440–8. 17942819 10.1212/01.WNL.0000265401.62434.36

